# Natives Against Invaders: Shared Use of Space and Temporal Segregation of Clouded Tiger‐Cats (*Leopardus pardinoides*) and Domestic Dogs (*Canis familiaris*) in an Isolated Protected Area

**DOI:** 10.1002/ece3.73770

**Published:** 2026-06-03

**Authors:** J. C. Cepeda‐Duque, A. M. López‐Barrera, E. Arango‐Correa, J. F. Hernández‐Fitzgerald, V. López‐Velasco, L. A. Fox‐Rosales, T. G. de Oliveira

**Affiliations:** ^1^ Tiger Cats Conservation Initiative São Luís Brazil; ^2^ Wildlife Conservation and Ecology Lab – Lab‐CEVS Universidade Estadual do Maranhão (UEMA) São Luís Maranhão Brazil; ^3^ Organización Ambiental Chinampa Risaralda Colombia; ^4^ Andean Bear Initiative Armenia, Quindío Colombia; ^5^ Max Planck Institute of Animal Behavior Radolfzell Germany; ^6^ Instituto Pró‐Carnívoros Atibaia, São Paulo Brazil; ^7^ IUCN, Species Survival Commission Cat Specialist Group Ittigen Switzerland

**Keywords:** activity, ecological modeling, invasive predator, threatened species

## Abstract

Domestic dogs are widely distributed and exert profound effects on ecosystems, through competition, disease transmission, and killing of native species. Nevertheless, little is known about how the presence of domestic dogs interacts with habitat structure and food availability in determining habitat use of native carnivores. We investigated how the habitat use and activity of an ecologically sensitive mesopredator, the clouded tiger‐cat (*Leopardus pardinoides*), is affected by habitat structure, prey availability, anthropogenic pressures, and dog ( *Canis lupus familiaris* ) activity in an isolated protected area of the Colombian Andes. We collected data on the occurrence and activity of both species from 23 single‐camera traps placed in the cloud forest of the Alto del Nudo Soil Conservation District between July 2022 and September 2023. We used negative binomial generalized linear models to assess the habitat use of clouded tiger‐cats and dogs in relation to habitat structure (topography, vegetation productivity, and connectivity), prey availability (for clouded tiger‐cats), and human pressure (human modification and road proximity). We used kernel density functions and diel niche models to assess temporal relationships of clouded tiger‐cats with dogs and small mammals. Clouded tiger‐cat habitat use increased with topographic slope, but decreased with human modification. Dog detections did not affect clouded tiger‐cat habitat use, being detected in 100% of the sampling stations with clouded tiger‐cat presence (ratio of 4:1 dog‐clouded tiger‐cat detections). There was a low temporal overlap between both species, with dogs being mainly diurnal and clouded tiger‐cats nocturnal. Human modification and widespread invasion of dogs can negatively affect clouded tiger‐cats in this degraded protected area, but they reduce these pressures using well‐preserved forest at steep slopes in the night. We advise taking actions to reduce the spatial contact between clouded tiger‐cats and dogs through banning the unsupervised incursion of dogs into the protected area while implementing neutering and adoption campaigns.

## Graphical abstract

We studied the spatial and temporal relationships between clouded tiger‐cats and dogs in an isolated protected area of Colombia. Dogs influenced the activity patterns of clouded tiger‐cats but not their habitat use. Conservation actions should be directed to mitigate potential contact between both species.

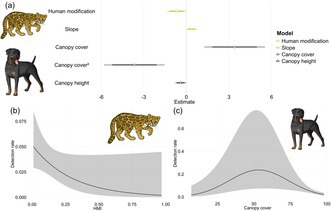

## 1 Introduction

Domestic dogs ( *Canis lupus familiaris* , hereafter, dogs) are the most abundant species of the order Carnivora on Earth, with a total biomass similar to that of all wild terrestrial mammals combined (Greenspoon et al. 2023). Dogs are social and cursorial predators that rely primarily on human‐derived food and safety, given their shared evolutionary history with humans (Gompper 2021; Hughes and Macdonald 2013). Dogs can compete, attack and transmit fatal infectious diseases to native wildlife and often hybridize with their native relatives (Doherty et al. 2017; Hughes and Macdonald 2013). When free ranging, they are able to disturb wildlife to a greater degree than native predators, even at the core of protected areas (Gompper 2015, 2021). Moreover, with the pet industry sustaining > US$200 billion in the global economy of an ever‐growing human pet‐dependent population, it is unlikely that any wildlife species will be free from interacting with dogs in the future (Bryce 2021). Dogs have caused 11 vertebrate extinctions (mainly birds) and represent a confirmed or potential threat to 188 imperiled species, but these numbers are thought to be underestimated (Doherty et al. 2017).

Despite the well‐recognized ecological impacts that dog invasions represent to native mammal species worldwide, several knowledge gaps remain unfilled, especially in biodiverse regions of conservation concern (Díaz et al. 2023; Orduña‐Villaseñor et al. 2023). However, for the Northern Andes, there is emerging evidence on the effects of dogs on native carnivore populations (Díaz et al. 2023; Zapata‐Ríos and Branch 2016, 2018) and increasing research interest on this regard for protected areas of Colombia (Bedoya‐Durán et al. 2021; Cepeda‐Duque, Arango‐Correa, López‐Velasco, et al. 2024; Rodríguez‐León and López‐Arévalo 2019). Since the last century, the rampant human population growth in Colombia has catalyzed the land use change, gradually relegating montane cloud forests to steep terrain that is unsuitable for agricultural activities or urban development (Rodríguez et al. 2013). In this context, the role of protected areas is compromised given the increased exposure to edge effects and the subsequent invasions by domestic dogs with uncertain ownership status (Cepeda‐Duque, Arango‐Correa, Frimodt‐Møller, and Lizcano 2024; Hughes and Macdonald 2013; Yen et al. 2019; Young et al. 2011). Therefore, obtaining accurate evidence on the impact of dogs on wildlife is required in order to inform management actions, which are often context‐dependent and socially sensitive (Biswas et al. 2024; Contreras‐Abarca et al. 2022; Gompper 2021).

The clouded tiger‐cat (*Leopardus pardinoides*) is a small, nocturnal predator (2.3 kg) of the highland ecosystems (> 1500 up to 3690 masl) in the northern Andes and Talamanca mountain ranges of South and Central America, respectively (de Oliveira et al. 2024). Populations of this small cat in Colombia are mainly associated with montane cloud forests at middle to high elevations (2000–3000 masl), in areas distant from human settlements, with low occurrence of intraguild predators and high prey availability (Cepeda‐Duque, Andrade‐Ponce, et al. 2023). Clouded tiger‐cats play an important ecological role in controlling small mammal populations and are considered sentinel species given their vulnerability to human‐driven ecosystem change (Marneweck et al. 2022; Cepeda‐Duque, Andrade‐Ponce, et al. 2023). Listed as “Vulnerable” in the IUCN Red List of Threatened Species (under *L. tigrinus* ), clouded tiger‐cats face multiple threats, such as negative interactions with invasive species, habitat loss, road kills, illegal poaching, and trade (sensu Payán and de Oliveira 2016). Nonetheless, a reassessment of its IUCN Red List status is necessary, given the recent taxonomic separation of the clouded tiger‐cat from the *L. tigrinus* species complex (see de Oliveira et al. 2024 for more details). This new species status on the clouded tiger‐cat magnifies the implications of the significant declines in demographic trajectory and geographic range in face of the multiple threats this wild cat is experiencing worldwide (de Oliveira et al. 2024; Lescroart et al. 2023). Additionally, little is known about the abiotic and biotic drivers that enable the persistence of clouded tiger‐cats in a landscape (but see Cepeda‐Duque, Andrade‐Ponce, et al. 2023; Ramírez‐Fernández et al. 2024). Moreover, basic information on the effects of dogs on the habitat use and activity patterns of this elusive felid is lacking in important areas for regional conservation in the Central Andes of Colombia.

Here, we investigated the habitat use and activity of clouded tiger‐cats and dogs to understand their ecological interactions in an isolated protected area of the Central Andes of Colombia. We analyzed the habitat use of both species in relation to topography (slope and aspect), human pressures (human modification and road proximity), and habitat structure (canopy cover, canopy height, and forest landscape integrity). We also assessed the effects of the dog and the small (< 1 kg) mammal encounter rate (i.e., prey availability) on the habitat use of clouded tiger‐cats. We expected the habitat use of clouded tiger‐cats to be positively related to forests with high landscape integrity, closed and tall canopies, high prey availability and flat terrain. These conditions reflect well‐preserved habitats with minimal energetic costs in movement and with opportunities for hunting and avoiding predators (Cepeda‐Duque, Andrade‐Ponce, et al. 2023). Consequently, we expected the habitat use of clouded tiger‐cats to be negatively related to sites with high human modification, as this species is sensitive to human pressures (Cepeda‐Duque, Andrade‐Ponce, et al. 2023). We expected that clouded tiger‐cat habitat use would be negatively affected by dog encounter rates given the risk associated with coming into contact (e.g., mortality, lower access to resources, and spread of disease, among others, Weston and Stankowich 2015).

We expected that dog habitat use will be positively related to sites with high human modification and closer proximity to roads given their reliance on accessible human‐derived resources (Paschoal et al. 2018; Ribeiro et al. 2019; Soultan et al. 2021; Zanin et al. 2019). Conversely, we expect dog habitat use to be negatively affected by canopy cover, canopy height, and forest landscape integrity given decreased availability of human‐derived resources and increased potential of antagonistic encounters with other predators (Bianchi et al. 2020; Frigeri et al. 2014; Malhotra et al. 2021; Ribeiro et al. 2019). We also expected increased dog habitat use in sites with flat terrain to reduce energy expenditure and improve hunting grounds (given their chasing behavior on wildlife) when moving inside the protected area (Stobo‐Wilson et al. 2020).

Regarding activity patterns of both species, we expected that clouded tiger‐cats would exhibit a low degree of temporal overlap with dogs, given increased risks of attacks and interference during hunting, mate searching, and cub rearing activities (Avendaño‐Díaz et al. 2025; Bianchi et al. 2020; Cepeda‐Duque, Arango‐Correa, López‐Velasco, et al. 2024). We also expected that clouded tiger‐cat would exhibit high temporal overlap with small mammal prey, since this represents greater chances for resource intake (Botts, Eppert, Wiegman, Botts, et al. 2020; Cepeda‐Duque, Arango‐Correa, López‐Velasco, et al. 2024; Marinho et al. 2018). We will compare the temporal relationships of clouded tiger‐cats with dogs and small mammal prey between rainfall seasons assuming that precipitation seasonality influences resource and risk availability (Cepeda‐Duque, Arango‐Correa, López‐Velasco, et al. 2024). We expected temporal overlap of clouded tiger‐cats with dogs and small mammal prey to differ between working and resting days. Tourism intensification in resting days can increase human and dog activity inside protected areas and consequently disrupt native predator behavior and hunting success (Frigeri et al. 2014; Reilly et al. 2017; Van Scoyoc et al. 2023; Weston and Stankowich 2015).

## 2 Materials and Methods

**FIGURE 1:**
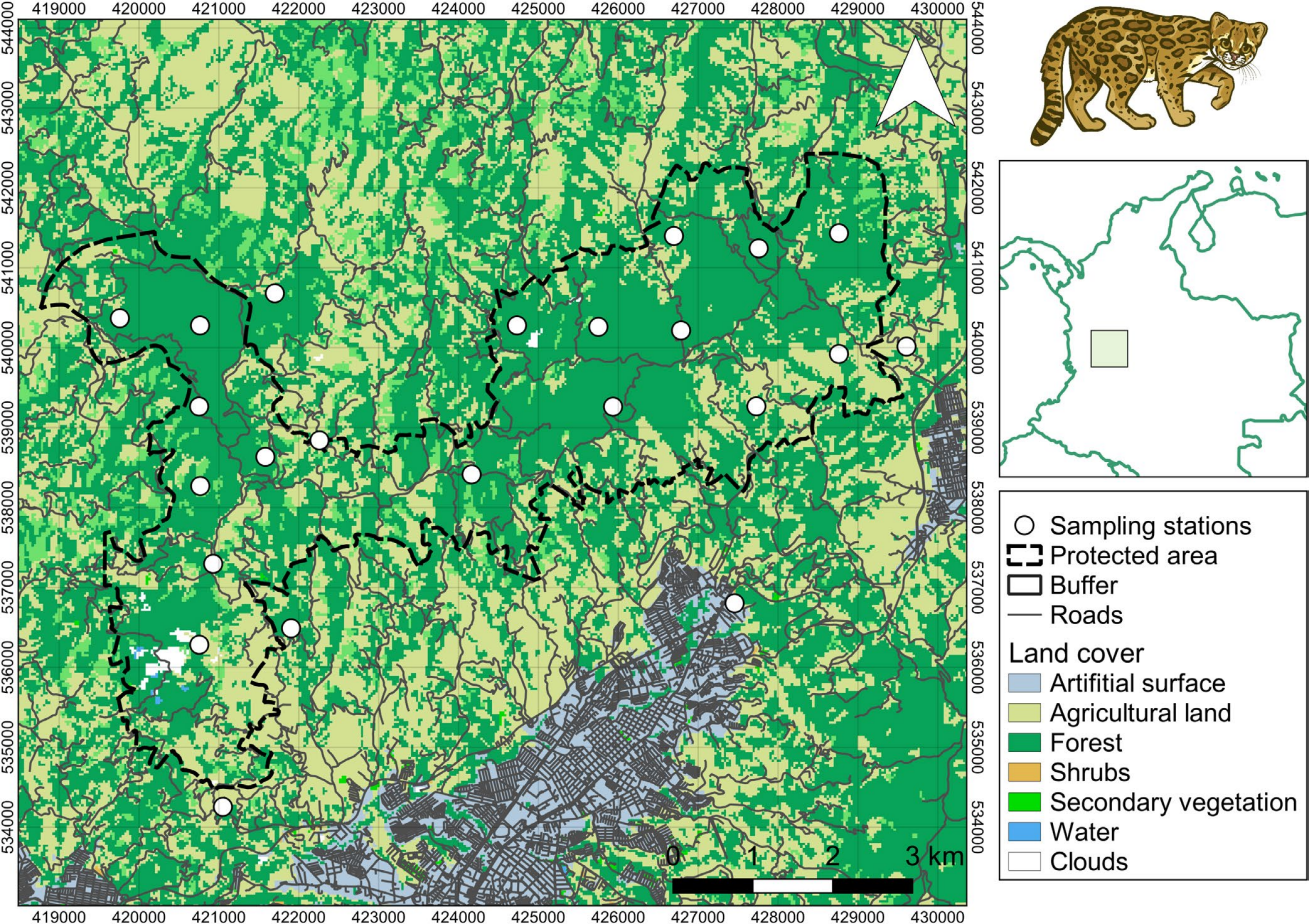
Camera trap survey was carried out between July 2022 and September 2023 in the Alto del Nudo Soil Conservation District to determine the habitat use and activity of clouded tiger‐cats in relation to habitat features, prey availability, and human pressures. Land cover was obtained from González‐González et al. (2022), with artificial surface referring to urban areas; the vector image of clouded tiger‐cat is from our own sources; the vector of roads was obtained from the online service of the Departamento Administrativo Nacional de Estadística (DANE – https://geoportal.dane.gov.co/servicios/descarga‐y‐metadatos/descarga‐mgn‐marco‐geoestadistico‐nacional/2022).

We conducted this study in two contiguous protected areas: Alto del Nudo Soil Conservation District and La Nona Protective Forest Reserve (hereafter ANSCD). The ANSCD is located in the western foothills (4.876412, −75.684802) of the Central Andes of Colombia, south of the Risaralda Department (Figure 1). The elevation in the area ranges from 1500 to 2200 masl, with a mean annual temperature of 20°C, and a mean annual precipitation of 1480 mm with two rainy seasons: between March–May and October–December (Londoño et al. 2004). Four human settlements in Risaralda surround the ANSCD, the cities of Pereira (482483 inhabitants), and Dosquebradas (230086), and the towns of Santa Rosa de Cabal (81608), and Marsella (16716). At the highest elevations, the montane cloud forest is mainly formed by isolated patches in the hills of ANSCD and at lower elevations by riverine strips of forest. Other dominant land cover types include residential complexes, cattle ranching pastures, eucalyptus and pine plantations, and multiple types of crops, including avocado, coffee, and citrus fruits. There are several human pressures within the limits of ANSCD, including hunting, logging, intensive tourism, noise, and light pollution (Londoño et al. 2004).

Between July 2022 and September 2023, we installed single‐camera unbaited stations as close as possible to the centroids of a regular grid of 1 km^2^ cells overlapping the montane cloud forest cover present in the landscape. In these centroids, we selected sites based on habitat features that would maximize use by clouded tiger‐cats, while minimizing the possibility of vandalism (e.g., abundant leaf litter and forest cover, presence of animal trails, see Cepeda‐Duque, Andrade‐Ponce, et al. 2023; Ramírez‐Fernández et al. 2024). In each site, we fastened the cameras (Bushnell Trophy Cam, Blaze Video A262) to trees < 50 cm above the ground and cleared any obstructions 1–2 m of the detection field to reduce false triggers (Ramírez‐Fernández et al. 2024). We set the camera traps to have the fastest trigger speed (1 s for Bushnell and 0 s for Blaze Video), the highest PIR sensitivity, and to capture at least three picture sequences. We checked the array of camera traps every 3 months to replace the batteries, SD cards, and cameras when needed. None of the cameras were stolen during the study period and excepting the research team; no humans were detected. One camera experienced malfunction (recording only for 14 days); therefore, we decided to exclude it from the analysis.

### 2.1 Covariates

To infer the habitat use of clouded tiger‐cats and dogs, we used the following predictor variables: proximity to roads, proximity to human settlements, Human Modification Index (HMI), Forest Landscape Integrity Index (FLII), dog and small mammal encounter rates (for clouded tiger‐cats), canopy cover and canopy height, elevation, aspect, and slope. We calculated the encounter rates as the number of dog and small mammal prey (shrews, marsupials, and rodents < 1 kg) detections (all pictures taken at least 1 h apart) divided by the sampling effort (number of days with camera activity) per sampling station (O'Brien et al. 2003). We calculated the nearest distance between roads and human settlements against the sampling stations using the NNJoin plugin of QGIS version 3.22.7 (Cepeda‐Duque, Andrade‐Ponce, et al. 2023). We used the QGIS plugin Point Sampling Tool to extract the most updated values at the finest spatial resolution possible from the following raster layers: HMI (300 m^2^ grids, Theobald et al. 2025), FLII (300 m^2^ grids, Grantham et al. 2020), canopy cover (30 m^2^ grids, Hansen et al. 2013), and canopy height (30 m^2^ grids, Potapov et al. 2021). The HMI weighs the cumulative effects of all the anthropogenic threats from the IUCN (https://www.iucnredlist.org/resources/threat‐classification‐scheme) in historical (1990–2020) and recent (2022) times (Theobald et al. 2025). The HMI scores range from unmodified or “wild” (0.0) to highly modified or developed (1.0) (Theobald et al. 2025). The FLII weighs the cumulative effects of observed and inferred pressures with the dynamics of forest connectivity in a landscape (Grantham et al. 2020). The FLII scores range from low (0) to high (10) forest integrity (Grantham et al. 2020). We conducted a multicollinearity inspection in all the numerical predictors through a Pearson correlation plot and a variance inflation factor (VIF) with a VIF > 3 indicating collinearity. Based on an established protocol, we recalculated the VIF values while dropping predictors with the highest VIF and interpreting model parameters in each drop until reaching the selected VIF threshold in the remaining predictors (Santon et al. 2023; Zuur et al. 2010). The VIF values were calculated with the package vegan 2.7‐1 (Oksanen et al. 2020).

Accordingly, two predictors presented collinearity and were removed from posterior analyses; elevation was negatively correlated with HMI and distance to human settlements was positively correlated with distance to roads (Figure S1). We decided to exclude elevation from the modeling for two reasons: first, the sampling stations covered a limited elevation range (1525–2136 masl) compared to the clouded tiger‐cat elevation range (540–3960 masl, de Oliveira et al. 2024). Second, conservation implications of testing the underlying hypothesis of clouded tiger‐cat habitat use and the effects of human pressures alone or additive to dog encounter rates. We excluded distance to human settlements from posterior analysis given its correlation with distance to roads, including the fact that HMI is not correlated with the same predictor but is way more informative than distance to human settlements alone (Figure S1). We standardized all the numerical covariates to have a mean of 0 and a standard deviation of 1 to improve model convergence (Brooks et al. 2017; Santon et al. 2023). For details on the range of predictor and VIF values, as well as descriptive statistics, please refer to Table S1.

### 2.2 Habitat Use

We pooled raw detections of clouded tiger‐cats and dogs at 1‐h intervals to avoid biases due to temporal autocorrelation (Cepeda‐Duque et al. 2021; Cepeda‐Duque, Arango‐Correa, López‐Velasco, et al. 2024; Lazzeri et al. 2022; Smith 2025). We then analyzed the habitat use of clouded tiger‐cats and dogs through generalized linear models (GLM) using independent detections of both species as response variables against a set of competing models following a priori hypotheses on predictor relationships (Aurich‐Rodriguez et al. 2022; Ferretti et al. 2023). We considered the GLM modeling approach desirable since the survey period (July 2022–September 2023) violated the closure assumption required to run occupancy or co‐occurrence models (Aurich‐Rodriguez et al. 2022; DiRenzo et al. 2022; Fennell et al. 2023; Lonsinger 2022). Hence, we assume that encounter rates are reliable indicators of clouded tiger‐cat and dog habitat use in the study area and that any potential variation in detection rates will be explained by the selected predictors (Mandujano 2024; Fennell et al. 2023; O'Brien 2011). We used the sampling effort as an offset term in our response variable (number of detections) to correct for differences in the performance of the sampling stations.

We run the models under a negative‐binomial probability distribution since it is more flexible to overdispersed count variables (Aurich‐Rodriguez et al. 2022; Fennell et al. 2023; Granados et al. 2023). We used the Akaike's information criterion corrected for small sample sizes (AICc) to rank models and determine parameter importance for each model (Burnham and Anderson 2002). First, we defined the null structure of our clouded tiger‐cat and dog habitat use models to assess (1) unaccounted variability using sampling stations as random effects, (2) the variance structure of the negative binomial distributions (quadratic vs. linear), and (3) the inclusion or removal of a zero‐inflation parameter. Once we defined the null model structure for clouded tiger‐cats and dogs, we built models with univariate relationships with the selected predictors, as our limited number of sampling stations can induce overfitting with more complex models (Sousa‐Guedes et al. 2026). However, we made an exception with one clouded tiger‐cat model including the additive effects of dog encounter rates and HMI, and two dog models including the quadratic effects of canopy cover and canopy height. We performed the clouded tiger‐cat additive model because we wanted to test whether the effects of dog encounter rates on the species' habitat use varied across a human modification gradient (Malhotra et al. 2021). We also tested the quadratic effect of canopy cover and canopy height on dogs to account for potential non‐linear relationships with vegetation, as recommended for ecologically flexible canids (Allen et al. 2025; Reasoner et al. 2024).

We inspected the estimated *β* coefficients from the top‐ranked model and we did not consider a variable informative if the 85% and 95% confidence intervals (CI) of the *β* coefficient crossed 0 (Santon et al. 2023; Sutherland et al. 2023). We screened model residuals and spatial autocorrelation (Moran's *I*) in the top‐ranked models (Aurich‐Rodriguez et al. 2022; Cepeda‐Duque, Arango‐Correa, et al. 2023; Ferretti et al. 2023). We used the packages glmmTMB version 1.1.11 (Brooks et al. 2017) to run the negative binomial models, MuMIn version 1.48.11 to select the top‐ranked models (Bartoń 2025), and DHARMa version 0.4.7 (Hartig 2024) to inspect mode assumptions of the residuals, respectively. All the analysis were run through the R Software (R Core Team 2024). For the detailed information regarding the model residual inspection, refer to the Figures S2–S6.

### 2.3 Activity Patterns

We transformed time data of the independent detections for clouded tiger‐cats, small mammal prey, and dogs from conventional time to decimal hours to apply circular statistics and test for uniformity in their activity patterns during the 24‐h cycle (Avendaño 2019). We produced 95% CI for the circular data of clouded tiger‐cats, small mammal prey, and dogs using 1000 bootstrap replicates (Avendaño 2019; Cepeda‐Duque, Arango‐Correa, López‐Velasco, et al. 2024). We tested whether the null hypothesis of species activity was uniformly distributed during the day using the Hermans–Rasson uniformity test with 1000 bootstrap replicates for the whole survey, for each rainfall season, and for working and resting days (Landler et al. 2019). The Hermans–Rasson test is preferred over other uniformity tests since it is sensible to asymmetric and multimodal activity curves (Landler et al. 2019).

We created activity curves for clouded tiger‐cats, small mammal prey, and dogs using non‐parametric kernel density functions with smoothed parameters of 50% and 95%, reflecting their core and range activities, respectively (Oliveira‐Santos et al. 2013). These parameters incorporate inordinate peaks in activity emerging from different criteria to group multiple detections into a single observation (Oliveira‐Santos et al. 2013). We then estimated the overlap coefficient between the activity curves of clouded tiger‐cats with those of small mammal prey and dogs, as it reflects the proportion of the larger activity curve shared by the smaller activity curve (Ridout and Linkie 2009). This coefficient ranges between 0 and 1, where 0 indicates no overlap and 1 indicates a complete overlap (Cepeda‐Duque, Arango‐Correa, López‐Velasco, et al. 2024; Ridout and Linkie 2009). We estimated the overlap for the smoothed parameters and a mean overlap derived by 1000 bootstrap replicates with 95% confidence intervals (Cepeda‐Duque et al. 2021; Cepeda‐Duque, Arango‐Correa, López‐Velasco, et al. 2024) for clouded tiger‐cat activity curves in relation to dogs and small mammals. To determine whether there were differences in the distribution of activity records during the 24‐h cycle of clouded tiger‐cats against dogs and potential prey, we performed a Watson two‐sample test (Cepeda‐Duque, Arango‐Correa, López‐Velasco, et al. 2024; Ferretti et al. 2023).

To assess whether the overlap coefficients of clouded tiger‐cats with small mammal prey and dogs differed in sites with varying levels of dog presence, we divided the stations into values with high and low dog encounter rates (Ho et al. 2025). Given the skewed distribution of dog encounter rates (Table S1), we split stations in values above and below the 50th percentile instead of the mean to ensure a balanced comparison in clouded tiger‐cat activity between sites with high versus low dog presence, respectively. This resulted in 13 stations with high dog encounter rates and 10 stations with low dog encounter rates. We also assessed whether the temporal overlap of clouded tiger‐cats with small mammal prey and dogs differed among different human activities during the week by dividing the activity events according to working (weekdays) and resting days (weekends and holidays).

We assessed whether clouded tiger‐cats and dogs maximized their activity during nighttime, daytime, or twilight hours using the modeling approach proposed by Gerber et al. (2024). This approach compares diel niche hypotheses using Bayes factors and estimates model parameters using a multinomial model with linear inequality constraints (Gerber et al. 2024). We delimited each diel period based on the mean solar time of the sunrise and sunset for all the detections of both species. We defined the twilight period as 1 h before sunrise (06:01 ± 00:02 SD) and 1 h after sunset (18:14 ± 00:01 SD). Consequently, we defined the nighttime (19:14–05:00) and daytime (06:02–18:13) diel periods and then proceeded to calculate the number of detections of both species within each diel period. We estimated the Bayesian posterior probability with 95% credibility intervals for each species using a particular diel period of the day more than the remaining periods (Gerber et al. 2024). We used three MCMC samplers with 5000 iterations and discarded 1000 iterations as burn‐in.

We used the *overlap* package version 0.3.9 (Ridout and Linkie 2009) to estimate the overlap coefficient, the *circular* package version 0.4‐97 (Agostinelli and Lund 2022) for the circular metrics and the Watson two‐sample test, and the *CircMLE* package version 4.5.1 (Landler et al. 2019) to estimate the Hermans–Rasson test. We used the *suncalc* package version 0.5.1 (Thieurmel and Elmarhraoui 2019) to obtain the mean solar time of the sunrise and the sunset. We used the *Diel.Niche* package version 0.0.1 to estimate the Bayesian posterior probabilities for each time period according to the “Maximizing” hypothesis, as it is optimal to understand intraspecific mortality risk in wild cats (Allen et al. 2024).

## 3 Results

From the 4657 trap‐nights, we obtained 367, 948, and 1366 raw images of clouded tiger‐cats, small mammal prey, and dogs, respectively. After collapsing the data into 1‐h intervals, we obtained 106, 305, and 218 clouded tiger‐cat, small mammal prey, and dog detections, respectively. Mean encounter rates were 3.24 ± 2.50 detections per 100 trap‐nights for clouded tiger‐cats, 8.7 ± 10.88 for small mammal prey, and 9.2 ± 8.47 for dogs. We obtained detections of clouded tiger‐cats and dogs in 74% and 87% of the sampling stations, respectively, with 73% of the sampling stations having detections of both species. When considering only stations with clouded tiger‐cat detections, the mean dog encounter rate was 11.29 ± 14.61 detections per 100 trap‐nights. Overall, dogs were detected approximately four times more frequently than clouded tiger‐cats, with an average ratio of 4 dog detections per clouded tiger‐cat detection (4:1). Dog records ranged from single individuals (100% of sampling stations with dog detections) to packs conformed by two (79%), three (37%), and four individuals (11%). We obtained only one detection of a puma ( *Puma concolor* ), the only top predator in the assemblage.

### 3.1 Habitat Use

**FIGURE 2:**
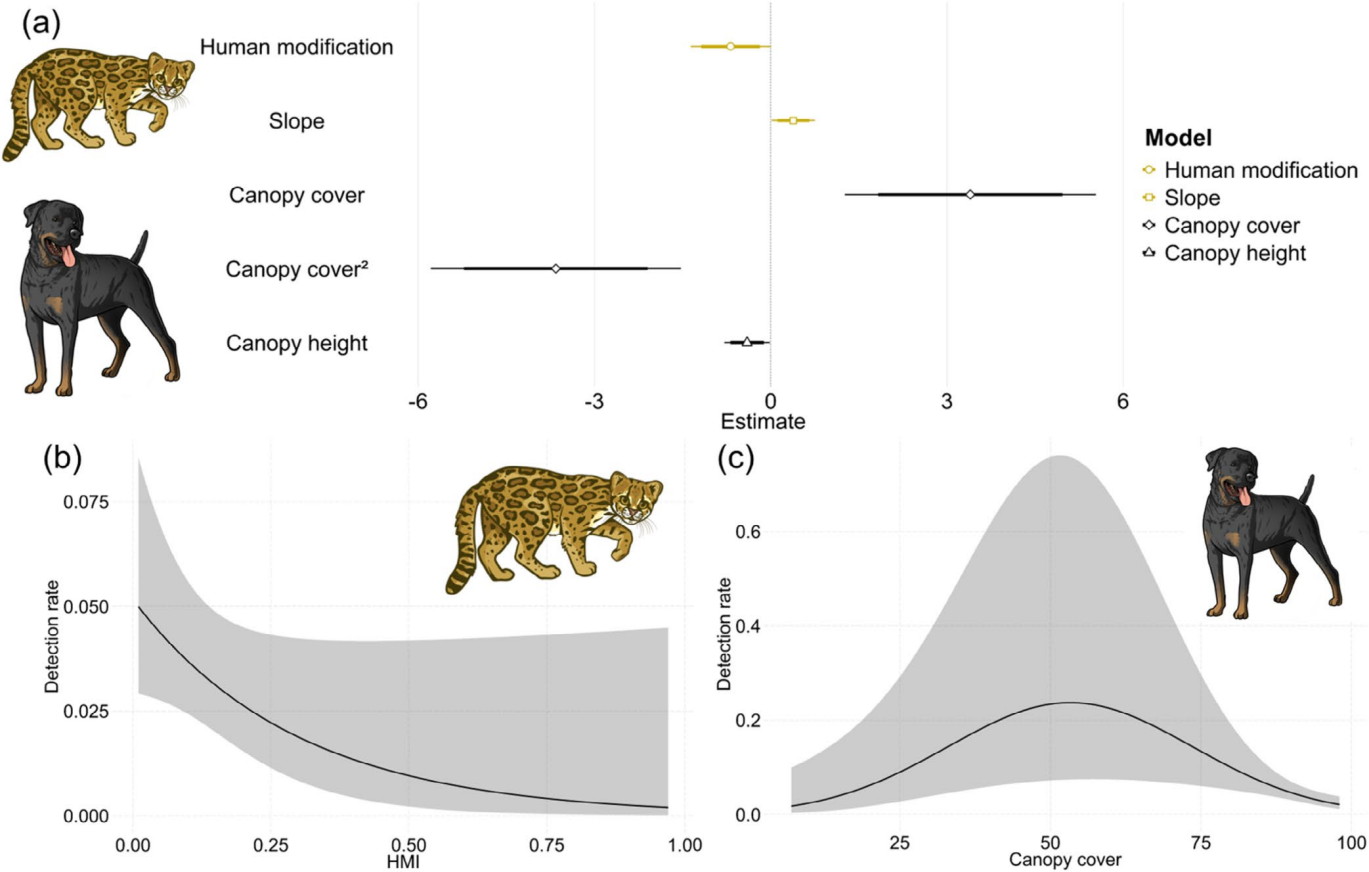
Effects of the relevant predictors in the habitat use of clouded tiger‐cats and dogs from the (a) inferential (e.g., effect size) and (b, c) predictive (AICc) perspectives. The habitat use of clouded tiger‐cats increased in well‐preserved forests (low Human Modification Index—HMI) at the steepest terrain (high slope), whereas the habitat use of dogs was higher in forests with intermediate canopy cover and smaller trees. The habitat use is represented as the detection rate (number of detections corrected by the sampling effort for each camera trap station per 100 trap‐nights). Values on the *x*‐axis are provided on the original scale for the HMI, slope (degrees), and canopy cover (percentage). For the inferential plot (a), we represented the 85% and 95% confidence intervals with thin and thick lines, respectively. For the predictive plots (b, c), we represented the 95% confidence intervals with a gray shading.

The null model used to understand clouded tiger‐cat habitat use was structured with a linear variance function and no random effects or zero‐inflation parameters (Table S2) and did not perform better than the models with predictors (Table 1). The best supported model in explaining clouded tiger‐cat habitat use included the effect of human modification (Table 1), with residual inspection showing good fit (Figures S2a–S6a) and no signs of spatial autocorrelation (Moran *I* = −0.09, *p* = 0.28). There was another highly ranked model which included the effect of slope on clouded tiger‐cat habitat use (Table 1) with residuals showing good fit (Figures S2b–S6b) and no spatial autocorrelation (Moran *I* = −0.09, *p* = 0.34). Accordingly, these models indicated that clouded tiger‐cat habitat use increased in sites with low human modification (Figure 2a, Table 2) and with high terrain slopes (Figure 2b, Table 2). Contrary to our expectations, neither prey availability nor dog encounter rates affected clouded tiger‐cat habitat use, and this was consistent either when dog encounter rates were modeled alone, or as additive to human modification (Table 1, Table 2).

**TABLE 1:** Model selection for the clouded tiger‐cat and dog habitat use models of a camera trap survey conducted between July 2022 and September 2023 in the Alto del Nudo Soil Conservation District.

Model	df	logLik	AICc	AIC_∆_	AIC_w_
**Clouded tiger‐cat**					
HMI	3	−57.756	122.8	0	0.482
Slope	3	−58.952	125.2	2.39	0.146
HMI + dog ER	4	−57.505	125.4	2.52	0.137
Null	2	−60.996	126.6	3.78	0.073
Canopy height	3	−60.44	128.2	5.37	0.033
Rodent ER	3	−60.522	128.4	5.53	0.03
Canopy cover	3	−60.557	128.4	5.6	0.029
FLII	3	−60.588	128.5	5.66	0.028
Aspect	3	−60.881	129.1	6.25	0.021
Dog ER	3	−60.889	129.1	6.27	0.021
**Dog**					
Canopy cover^2^	4	−66.286	142.9	0	0.643
Canopy height	3	−69.617	146.6	3.64	0.104
Null	2	−71.434	147.5	4.57	0.065
Canopy height^2^	4	−68.826	148	5.08	0.051
Slope	3	−70.42	148.2	5.25	0.047
FLII	3	−70.648	148.6	5.7	0.037
Distance to roads	3	−71.347	150	7.1	0.018
Aspect	3	−71.367	150.1	7.14	0.018
HMI	3	−71.432	150.2	7.27	0.017

Abbreviations: AIC_∆_, AICc difference; AICc, Akaike information criterion for small sample sizes; AICw, AIC weight; Df, Degrees of freedom; ER, Encounter rates; FLII, Forest Landscape Integrity IndexHMI, Human Modification Index; logLik, Log‐likelihood.

**TABLE 2:** Regression coefficients, standard error (SE), and confidence intervals of the model parameters analyzed to understand the habitat use of clouded tiger‐cats and dogs between July 2022 and September 2023 in the Alto del Nudo Soil Conservation District.

Model	Parameter	β	SE	Lower (85%)	Upper (85%)	Lower (95%)	Upper (95%)
**Clouded tiger‐cat**							
HMI	Intercept	−3.534	0.226	−3.861	−3.207	−3.979	−3.089
	HMI	−0.683	0.347	−1.183	−0.183	−1.364	−0.002
Slope	Intercept	−3.463	0.217	−3.776	−3.151	−3.888	−3.038
	Slope	0.385	0.188	0.114	0.655	0.017	0.753
HMI + dog ER	Intercept	−3.529	0.223	−3.864	−3.193	−3.986	−3.071
	HMI	−0.744	0.381	−1.293	−0.195	−1.492	0.004
	Dog ER	0.177	0.223	−0.144	0.499	−0.260	0.615
Null	Intercept	−3.389	0.221	−3.707	−3.071	−3.822	−2.956
Canopy height	Intercept	−3.428	0.224	−3.751	−3.105	−3.868	−2.988
	Canopy height	0.195	0.184	−0.070	0.460	−0.165	0.556
Rodent ER	Intercept	−3.427	0.223	−3.749	−3.105	−3.865	−2.989
	Rodent ER	0.180	0.176	−0.074	0.434	−0.165	0.526
Canopy cover	Intercept	−3.392	0.215	−3.702	−3.082	−3.814	−2.970
	Canopy cover	−0.189	0.182	−0.451	0.073	−0.546	0.168
FLII	Intercept	−3.429	0.231	−3.762	−3.095	−3.883	−2.975
	FLII	0.226	0.264	−0.155	0.607	−0.292	0.744
Aspect	Intercept	−3.385	0.218	−3.700	−3.070	−3.813	−2.956
	Aspect	−0.097	0.202	−0.389	0.195	−0.495	0.300
Dog ER	Intercept	−3.376	0.224	−3.700	−3.052	−3.817	−2.935
	Dog ER	0.116	0.232	−0.218	0.450	−0.339	0.571
**Dog**							
Canopy cover^2^	Intercept	−3.264	0.189	−3.536	−2.992	−3.635	−2.893
	Canopy cover	3.398	1.090	1.828	4.967	1.261	5.535
	Canopy cover^2^	−3.658	1.086	−5.222	−2.094	−5.787	−1.528
Canopy height	Intercept	−3.101	0.209	−3.403	−2.800	−3.512	−2.691
	Canopy height	−0.402	0.197	−0.686	−0.117	−0.789	−0.014
Null	Intercept	−3.026	0.225	−3.351	−2.701	−3.469	−2.583
Canopy height^2^	Intercept	−3.132	0.204	−3.426	−2.838	−3.533	−2.732
	Canopy height	−1.930	1.261	−3.745	−0.115	−4.402	0.542
	Canopy height^2^	1.517	1.228	−0.252	3.285	−0.891	3.925
Slope	Intercept	−3.074	0.218	−3.388	−2.760	−3.501	−2.647
	Slope	0.405	0.284	−0.004	0.815	−0.152	0.963
FLII	Intercept	−3.059	0.218	−3.374	−2.744	−3.488	−2.631
	FLII	−0.314	0.253	−0.679	0.051	−0.811	0.183
Distance to roads	Intercept	−3.027	0.225	−3.352	−2.703	−3.469	−2.586
	Distance to roads	0.088	0.210	−0.215	0.391	−0.325	0.500
Aspect	Intercept	−3.027	0.225	−3.351	−2.702	−3.468	−2.585
	Aspect	−0.101	0.274	−0.496	0.294	−0.639	0.437
HMI	Intercept	−3.026	0.225	−3.351	−2.701	−3.469	−2.583
	HMI	0.023	0.375	−0.518	0.564	−0.714	0.759

*Note:* Confidence intervals are provided with a significance of 85% and 95%, respectively.

Abbreviations: ER, Encounter rate (number of detections divided by sampling effort and multiplied by 100 trap‐nights); FLII, Forest Landscape Integrity Index; HMI, Human modification Index.

The null model used for dog habitat use was structured with a quadratic variance function and no random effects or zero‐inflation parameters (Table S2) and did not perform better than the models with predictors (Table 1). The best supported model in explaining dog habitat use included the quadratic effect of canopy cover (Table 1), with residual inspection showing good fit (Figures S2c–S6c). In addition, the residuals of the top‐ranked model were not spatially autocorrelated (Moran *I* = −0.04, *p* = 0.72). Accordingly, dog habitat use initially increased with canopy cover to a moderate amount but then decreased in sites with high canopy cover (Figure 2c, Table 2). In addition, we found support for the effect of canopy height on dog habitat use, with decreased use of sites with tall canopies (Figure 2d), as indicated by the 85% and 95% confidence intervals below 0 (Table 2). The residual inspection indicated that this model also showed good fit (Figures S2d–S6d) excepting only for the quantile regression of the residuals (Figure S3d), suggesting some non‐linearity in the data that was not captured in the model structure. This model did not present signs of spatial autocorrelation (Moran *I* = −0.04, *p* = 0.77).

### 3.2 Activity Patterns

Overall, most of the clouded tiger‐cats and small mammal prey detections occurred during the nighttime (78% and 87% of the detections, respectively), followed by the twilight (14% and 10%), and the daytime (8% and 10%). Dogs were mostly diurnal (72% of records), with few detections during nighttime (16%) and twilight (12%). Clouded tiger‐cats and small mammal prey were consistently nocturnal during the rainfall seasons, between the periods of human activity and between sites with varied dog encounter rates (Table 3). Mean activity for clouded tiger‐cats was concentrated between 22:19 and 23:37, while for small mammal prey, it was concentrated between 22:44 and 00:26. The activity curves of clouded tiger‐cats and small mammal prey indicated the existence of two main peaks, before midnight and before sunrise (Figure S7). Dog diurnal activity was also consistent during rainfall seasons and periods of human activity, with mean activity concentrated mainly after midday, between 13:29 and 13:58 (Table 3). Mean activity of dogs was also concentrated after midday in sites with high dog encounter rates, but in sites with low dog encounter rates, the mean activity occurred later in the sunset (Table 3). The dog activity curves exhibited two main activity peaks, one highly concentrated after midday and another one less pronounced in the middle morning (Figure S8).

**TABLE 3:** Circular mean, deviation (SD), and 95% confidence intervals (CI) calculated for the activity patterns of clouded tiger‐cats, clouded tiger‐cat prey (small mammals), and dogs in the Alto del Nudo Soil Conservation District, Colombia.

Species	*N*	Mean (SD)	CI (95%)	HR	*p*	Category
Clouded tiger‐cats	106	23:04 (01:17)	21:51–00:21	28.162	< 0.001	Total
	46	22:19 (01:18)	20:27–00:04	12.309	< 0.001	Rainy
	60	23:36 (01:15)	21:57–01:14	19.330	< 0.001	Dry
	41	22:27 (01:24)	20:47–00:28	13.841	< 0.001	Resting
	65	23:27 (01:15)	20:54–00:18	17.879	< 0.001	Working
	50	22:37 (01:09)	21:15–00:17	20.412	< 0.001	Low
	56	23:35 (01:24)	21:37–01:33	11.318	< 0.001	High
Small mammal prey	305	23:37 (00:54)	23:10–00:04	177.567	< 0.001	Total
	148	22:44 (00:54)	22:09–23:22	99.859	< 0.001	Rainy
	157	00:26 (00:51)	23:51–01:01	100.743	< 0.001	Dry
	207	00:08 (00:58)	23:13–01:04	51.272	< 0.001	Resting
	98	23:24 (00:52)	22:54–23:54	132.593	< 0.001	Working
	111	00:30 (00:51)	23:47–01:10	72.167	< 0.001	Low
	185	23:09 (00:54)	22:39–23:43	112.773	< 0.001	High
Dogs	218	13:38 (01:18)	12:48–14:25	57.626	< 0.001	Total
	109	13:48 (01:21)	12:31–14:55	28.490	< 0.001	Rainy
	108	13:29 (01:15)	12:21–14:31	30.837	< 0.001	Dry
	61	13:58 (01:24)	12:06–15:40	14.576	0.002	Resting
	157	13:32 (01:16)	12:32–14:28	44.865	< 0.001	Working
	39	16:07 (01:26)	13:11–18:01	13.456	< 0.001	Low
	182	13:15 (01:14)	12:23–14:03	53.719	< 0.001	High

*Note:* An alpha level of 0.05 was chosen for the Hermans–Rasson (HR) test. Data were subdivided according to rainfall seasons (rainy and dry seasons), human activity (resting and working days), and dog encounter rates.

The resampled and range activity overlap coefficients provide support for high temporal synchronization between clouded tiger‐cats and small mammal prey, remaining consistent among rainfall seasons, days of human activity, and different dog encounter rates (Table 4). However, we found that clouded tiger‐cats were not synchronized with some of the activity peaks of small mammal prey, as evidenced by the lower overlap coefficient in the core activity of both species (Table 4). For instance, as previously mentioned, one peak of small mammal prey activity occurred before midnight (19:46–22:16) and the other one before sunrise (00:48–04:28), but clouded tiger‐cats were mainly synchronized with the former, particularly during resting days (Figure S9). This mismatch between overlapping in the core activity of clouded tiger‐cats and prey was enforced by the significant differences in the Watson two‐sample test (Figure S8).

**TABLE 4:** Overlap coefficients and Watson two‐sample test for the activity patterns of clouded tiger‐cats, potential clouded tiger‐cat prey (small mammals), and dogs in the Alto del Nudo Soil Conservation District.

	Category	Δ (0.95)	Δ (0.50)	Δ Resampled	CI
Clouded tiger‐cats vs. dogs	Total	0.58	0.0001	0.42	0.29–0.27
	Rainy	0.46	0.0009	0.50	0.32–0.58
	Dry	0.38	0.001	0.40	0.26–0.48
	Low	0.55	0.27	0.55	0.35–0.66
	High	0.38	0.001	0.45	0.30–0.53
	Resting	0.45	0.01	0.49	0.30–0.59
	Working	0.40	0.001	0.42	0.27–0.49
Clouded tiger‐cats vs. small mammal prey	Total	0.84	0.60	0.79	0.73–0.88
	Rainy	0.82	0.74	0.72	0.63–0.84
	Dry	0.80	0.37	0.76	0.67–0.88
	Low	0.76	0.29	0.73	0.62–0.86
	High	0.78	0.58	0.73	0.64–0.84
	Resting	0.82	0.40	0.73	0.65–0.88
	Working	0.82	0.40	0.76	0.68–0.87

*Note:* The overlap coefficients for the smoothed parameters of 95% and 50% are represented as well as the mean overlap estimated from 1000 bootstrap replicates with their corresponding 95% confidence interval (CI). Comparisons were made based on rainfall (rainy vs. dry season), human activity (resting vs. working days), and dog encounter rates calculated for all the sampling stations (low vs. high based on the 50th quantile).

**FIGURE 3:**
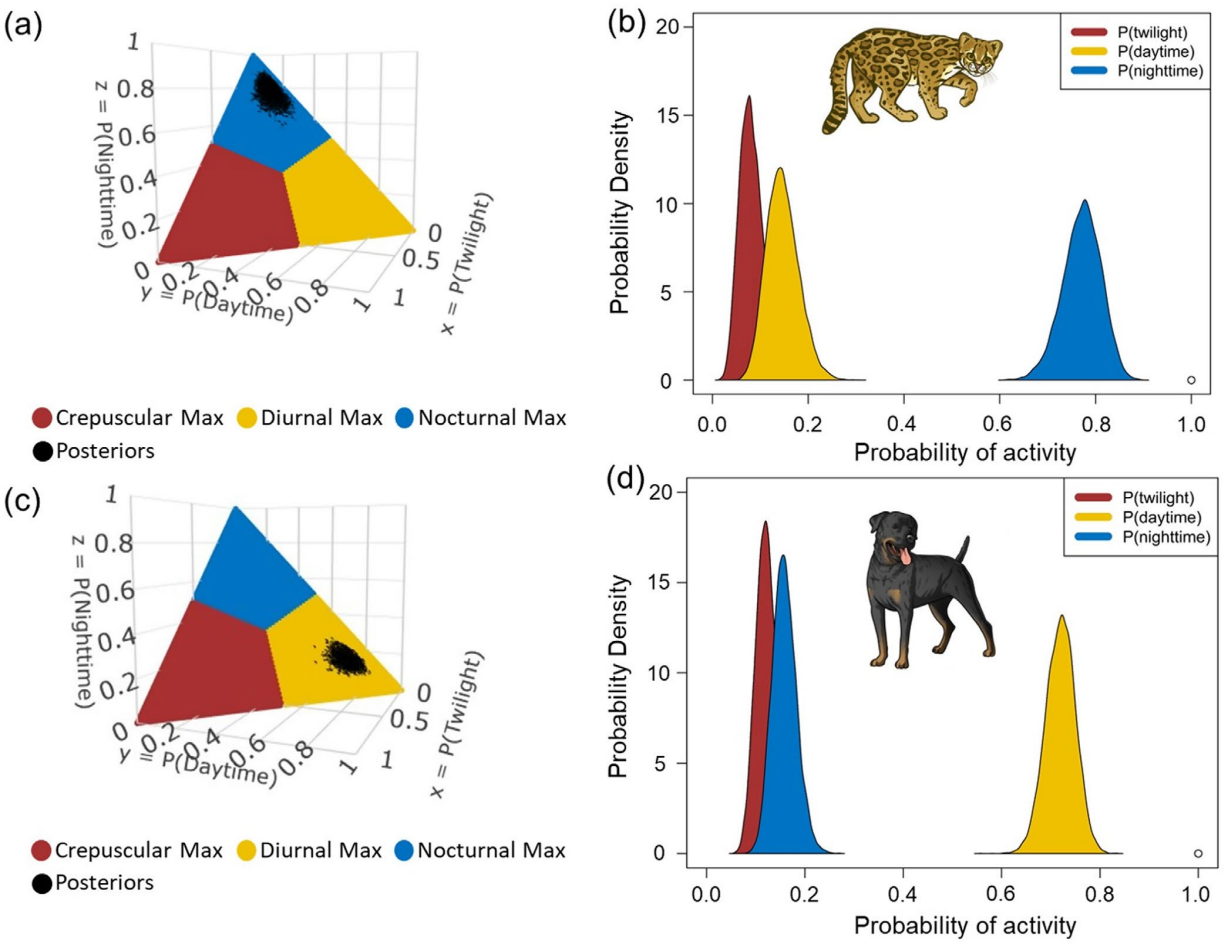
Posterior probabilities of activity (right) and the same information in three‐dimensional form (left), overlaying the maximizing hypothesis set for clouded tiger‐cats (a), (b) and dogs (c, d) in the Alto del Nudo Soil Conservation District between July 2022 and September 2023. The vector of clouded tiger‐cats is from our own sources, and the vector of dogs is from Canva.

**FIGURE 4:**
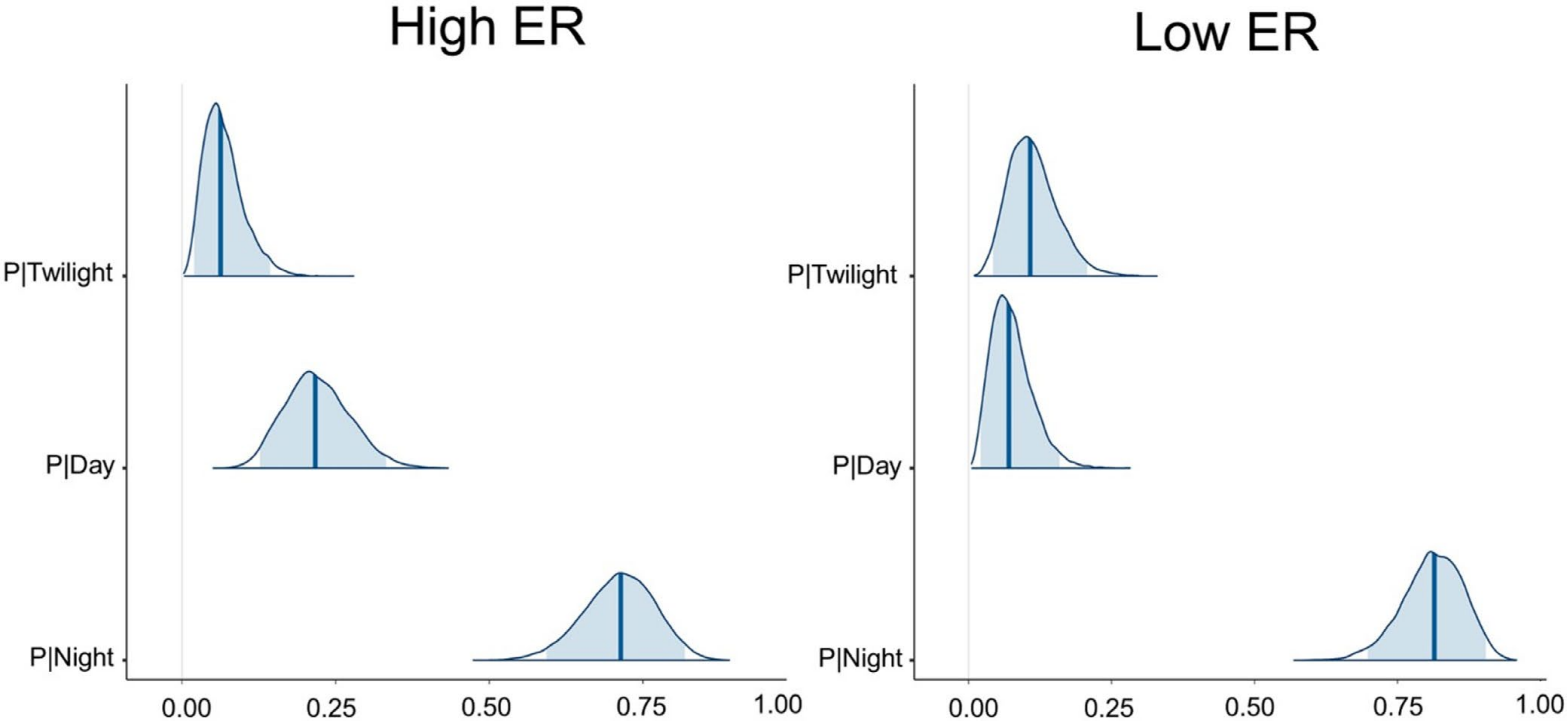
Posterior probabilities with medians and the 95% credibility intervals in the three diel periods for clouded tiger‐cats corresponding to sites with high (left, above the 50th quantile) and low (right, below the 50th quantile) dog encounter rates in the Alto del Nudo Soil Conservation District. We obtained the encounter rate of 2.69 detections of dogs per 100 trap/nights as the 50th quantile.

We found evidence of temporal segregation between clouded tiger‐cats and dogs, with both species exhibiting low overlap coefficients (Table 4). The overlap coefficient was lower during the dry season and at sites with higher dog encounter rates (Table 4). These results were more evident when comparing the overlap coefficient for the core activity with that obtained for the range activity (Table 4, Figure S9). In the case of the Watson two‐sample test, we also found significant differences in the pairwise comparisons of the activity curve distributions for clouded tiger‐cats with dogs (Figure S8). According to the diel niche models, we found high probabilities for clouded tiger‐cats to maximize nocturnal activity (Figure 3a,b, Table 5), independent of sites yielding high or low dog encounter rates (Figure 4). In contrast, we found high probabilities for dogs to maximize their activity during the daytime (Figure 3c,d, Table 5). The diel period with the lowest probability for clouded tiger‐cats and dogs was twilight (Table 5).

**TABLE 5:** Detection frequencies of clouded tiger‐cats and dogs in the Alto del Nudo Soil Conservation District and posterior probabilities with their corresponding 95% credibility intervals according to the “Maximizing” hypothesis set.

Parameter	Clouded tiger‐cats	Dogs
N daytime	15	158
N nighttime	83	34
N twilight	8	26
P | daytime	0.14	0.72
Lower CI daytime	0.08	0.65
Upper CI daytime	0.21	0.77
P | nighttime	0.77	0.15
Lower CI nighttime	0.68	0.11
Upper CI nighttime	0.84	0.20
P | twilight	0.07	0.12
Lower CI twilight	0.03	0.08
Upper CI twilight	0.14	0.16

Abbreviation: P, the posterior probabilities and CI the 95% credibility intervals.

## 4 Discussion

We provide insights into the habitat use and activity patterns of two predators with contrasting ecologies: clouded tiger‐cats (native, specialized and subordinate) and dogs (invasive, generalist and dominant), co‐occurring in an isolated protected area in the Central Andes of Colombia. Human modification of the landscape and topographic slope prevailed as the strongest predictors in explaining clouded tiger‐cat habitat use, underscoring their sensitivity to disturbances in the ANSCD landscape. In contrast, dog habitat use was mainly affected by vegetation structure, confirming their use of transitional habitats to spread across the protected area. We expected spatial and temporal avoidance between clouded tiger‐cats and dogs because the former can perceive dogs not only as competitors but also as intraguild predators (e.g., Granados et al. 2024; Ritchie et al. 2015; Vanak et al. 2015). Conversely, we found that dog encounter rates did not constrain clouded tiger‐cat habitat use. However, there was strong support for temporal segregation between both predators, with clouded tiger‐cats showing nocturnal activity in contrast to the diurnal activity of dogs. This study is novel in that it provides a comprehensive assessment of the effects of dogs on the spatiotemporal ecology of a newly recognized and already threatened small wild cat.

The lack of spatial avoidance between clouded tiger‐cats and dogs could be attributed to the widespread occurrence of the latter in the ANSCD, which was detected at every single camera trap station. This finding agrees with evidence obtained for savanna tiger‐cats ( *L. tigrinus* ) and ocelots (*L. pardalis*) in the Caatinga drylands of Brazil (Dias et al. 2019) and guiñas ( *L. guigna* ) in Chile (García et al. 2021; Malhotra et al. 2021). In contrast, previous studies on pampas (*L. colocola*) and Geoffroy's cats ( *L. geoffroyi* ) in the Mediterranean landscapes of Chile (Beltrami et al. 2023) and ocelots in the Atlantic Forest of Brazil suggest that these predators tend to avoid sites where dogs are present (Massara et al. 2018). This is commonly attributed to avoidance, as lethal encounters with dogs have been recorded in small wild cat species in South America, including guiñas, ocelots, jaguarundis (*Herpailurus yagouaroundi*), Geoffroy's cats, savanna tiger‐cats, and clouded tiger‐cats (Cepeda‐Duque, Arango‐Correa, López‐Velasco, et al. 2024; de Oliveira et al. 2020; Díaz et al. 2021; Pereira et al. 2010; Pinna and Magallanes 2020; Silva‐Rodríguez and Ortega‐Solís 2007). For instance, in the rural and protected areas of Argentina and Chile, a great amount of mortality of Geoffroy's cats (Pereira et al. 2010) and guiñas (Silva‐Rodríguez and Ortega‐Solís 2007), respectively, was attributed to dog attacks. Spatial segregation between clouded tiger‐cats and dogs may occur in the vertical habitat dimension, as the former has good climbing abilities (Weston and Stankowich 2015; Dymit et al. 2025; Zwicker et al. 2024). Small wild cats commonly climb trees to avoid encounters with dogs and other native predators, but also use this vertical habitat dimension to forage, rest, or nest (Beltrami et al. 2023; Bianchi et al. 2020; Dymit et al. 2025; Moreira‐Arce et al. 2015).

We found support for a negative relationship between clouded tiger‐cat habitat use and the HMI, suggesting that they could be sensitive to human‐derived pressures in isolated protected areas (Theobald et al. 2025). The HMI reflects the degree of urban development, agriculture, energy production, use of biological resources, human accessibility, modification of natural resources, pollution, and transportation (Theobald et al. 2025; Zarco‐González et al. 2025). All these factors combined can reduce the habitat quality and negatively impact carnivore habitat use, as has been found for black bears ( *Ursus americanus* ) in northeastern Mexico (Zarco‐González et al. 2025). Recent studies have shown the clouded tiger‐cat's preference for sites with low human disturbance, high forest cover (Ramírez‐Fernández et al. 2024), and advanced successional stages with a vertically complex microhabitat structure (Cepeda‐Duque, Andrade‐Ponce, et al. 2023). We obtained support for a strong and positive effect of topographic slope on the habitat use of clouded tiger‐cats. We suggest that clouded tiger‐cats could select steep terrain to have a greater chance of escaping lethal encounters with dogs and humans, without precluding access to resources. In the Trans‐Himalaya Mountain range, snow leopards ( *Panthera uncia* ) were strongly associated with steep slopes, not only to reduce competition with Tibetan wolves (*C. l. chanko*), dholes ( *Cuon alpinus* ) and dogs, but also to raise offspring in vital shelters (Justa and Lyngdoh 2023; Watts et al. 2019).

The increased habitat use of dogs in areas with poor canopy cover agrees with the notion of dog affinity for highly modified forests, which usually concentrate greater human activity when compared to dense forest (Frigeri et al. 2014; Paschoal et al. 2018; Ribeiro et al. 2019). Forests with dense canopy cover may be less accessible to humans and, consequently, unsuitable for dogs, since they not only spend more energy finding resources and defending territories, but are also more vulnerable to intraguild predation/competition (Biswas et al. 2024; Manzo et al. 2025). Transitional habitats can act as corridors for dogs to allow them to spread inside the limits of ANSCD given improved orientation and more rapid mobilization, which may also facilitate the conformation of dog packs (Ribeiro et al. 2019; Soultan et al. 2021). From another perspective, our results suggest that a more intact forest structure in the ANSCD can reduce clouded tiger‐cats and other wildlife exposure to dog attacks, disturbance and/or disease transmission (Malhotra et al. 2021; Ribeiro et al. 2019). Similar patterns have emerged in other tropical landscapes where dogs concentrate their activity in edge environments that provide both access to human resources and entry points into wildlife habitat (Dechner et al. 2018; dos Santos et al. 2017; Frigeri et al. 2014; Paschoal et al. 2012, 2018; Ribeiro et al. 2019). Dogs could be favored at sites with lower canopy heights because such features are prevalent in agricultural areas where dogs can be supplied by human‐derived resources (Paschoal et al. 2012, 2018). In two dog populations in Brazil, dogs were mostly associated with smaller trees and denser cover of cacao and rubber trees, coinciding with sites that are highly used by human workers (Dechner et al. 2018; Frigeri et al. 2014).

Clouded tiger‐cats were mostly nocturnal and highly overlapped with small mammals, in agreement with recent studies on the species's activity patterns elsewhere (Barrera‐Vargas et al. 2023; Botts, Eppert, Wiegman, Blankenship, et al. 2020; Cepeda‐Duque, Arango‐Correa, López‐Velasco, et al. 2024; Mooring et al. 2020). Nocturnal behavior in small Neotropical wild cats has been linked to synchronization with prey activities, as small mammals can be easily hunted in the dark (Botts, Eppert, Wiegman, Botts, et al. 2020; Cepeda‐Duque, Arango‐Correa, López‐Velasco, et al. 2024; Marinho et al. 2018). Moreover, lunarphobia in clouded tiger‐cats has been found to be related to greater synchronization with small mammal prey and improved avoidance of nocturnal intraguild predators (Cepeda‐Duque, Arango‐Correa, López‐Velasco, et al. 2024). However, recreational activities at protected areas can also induce nocturnal behavior in wild cats and even disturb predator–prey temporal relationships given increased perceived risks to encounters with humans (Fennell et al. 2023; Guzmán‐Aguayo et al. 2025; Reilly et al. 2017; Van Scoyoc et al. 2023). This could partially explain why we observed a decrease in temporal overlap between clouded tiger‐cats and small mammal prey before the sunrise during resting days, when tourism intensifies in the ANSCD. Nocturnal activity can also be linked to the avoidance of encounters with intraguild killers as a mechanism of coexistence when spatial segregation is unfeasible (de Oliveira and Pereira 2014; Zwicker et al. 2024). Our results in the ANSCD isolated protected area confirmed this, being consistent with the observed patterns for temporal segregation between clouded tiger‐cat and dogs in other protected cloud forests of the Central and Western Andes of Colombia (Cepeda‐Duque, Arango‐Correa, López‐Velasco, et al. 2024). Temporal segregation from dogs has also been recorded in small wild cats elsewhere in tropical forests (Carvalho et al. 2019; Horn et al. 2020; Malhotra et al. 2021; Pereda Sánchez et al. 2023; Weng et al. 2022). Nocturnality in clouded tiger‐cats, Peruvian desert cats (*L. garleppi*) and leopard cats ( *Prionailurus bengalensis* ) in sites with diurnal dogs were suggested to simultaneously reduce competition for prey and the risk of intraguild killing (Cepeda‐Duque, Arango‐Correa, López‐Velasco, et al. 2024; Pereda Sánchez et al. 2023; Weng et al. 2022). In contrast, other studies have found that small wild cats, such as Geoffroy's cats and margays ( *Leopardus wiedii* ), can display increased activity during daylight hours in sites intensely used by dogs (Boller et al. 2023; Zanón Martínez et al. 2022). Similarly, other species, such as jaguarundis and ocelots, showed moderate to high temporal overlap with dogs in several protected areas in the Cerrado and Atlantic Forest biomes in Brazil (Bianchi et al. 2020; Bolze et al. 2021).

Dogs were predominantly diurnal, consistent with reports from free‐ranging populations in protected areas elsewhere (Bianchi et al. 2020; Cepeda‐Duque, Arango‐Correa, López‐Velasco, et al. 2024; Justa and Lyngdoh 2023; Zanin et al. 2019; Zapata‐Ríos and Branch 2016). Diurnal activity in dogs is commonly associated with reliance on humans for food and safety, whereas feral individuals tend to be more nocturnal, likely to avoid human encounters (Bianchi et al. 2020; Griss et al. 2021; Zanin et al. 2019). The decrease in dog activity in the ANSCD forests around midday can be associated with dogs seeking shade close to humans to avoid thermal stress in roaming during the warmest daylight hours (Biswas et al. 2024; Maher et al. 2019). Beyond spending time with humans, dogs also engage in other behaviors which include patrolling territory, foraging, and mate search (Maher et al. 2019). For instance, free‐ranging dogs from India increased activity around the afternoon to enforce vigilance and territorial defense (Biswas et al. 2024). Even though nocturnal activity of dogs has also been documented in a nearby protected area to ANSCD, it was attributed to owned dogs from local residents ignoring restrictions, rather than truly feral dogs (Jiménez et al. 2017). The diurnal dog activity in the degraded ANSCD landscape is congruent for a mixed population of both owned and unowned dogs that facultatively conform packs while being supplied by human‐derived resources from the surrounding matrix. In this context, the proximity of ANSCD to several urban cores may facilitate the permanent recruitment of dogs either arriving on their own or being abandoned by humans (Yen et al. 2019).

### 4.1 Limitations and Future Directions

The modeling approach used to assess clouded tiger‐cat and dog habitat use may be insufficient to detect fine‐scale population effects of dogs on clouded tiger‐cats, as we did not account for imperfect detection (Mandujano 2024; Manzo et al. 2025; O'Brien 2011). Statistical co‐occurrence might not reflect true ecological interactions (Blanchet et al. 2020) since spatial overlap or segregation can arise from external drivers rather than ecological processes linked to predation or competition (Twining and Kellner 2025). Additionally, dogs were detected at all camera traps, precluding us from comparing sites with or without dogs. To better understand the effects of dogs on clouded tiger‐cats, we recommend modeling their population instead of photographic numbers while accounting for imperfect detection (Twining and Kellner 2025). As such, exploring the potential of co‐abundance models in assessing how these predators interact while incorporating the effects of relevant predictors seems a promising research avenue (Twining et al. 2024). For instance, potential for pack hunting can yield higher mortality rates and, while assisted by human subsides, can drive subordinate predators to exceed their carrying capacity leading to break downs in predator–prey regulation and decreased ecosystem integrity (Yen et al. 2019). Indirect effects of top predators can also modulate mesopredator interactions across the spatial and temporal niche dimensions (de Oliveira and Pereira 2014; Twining and Kellner 2025). Extending this study to landscapes with greater puma occurrence is needed to determine if they play a mediating role in clouded tiger‐cat and dog interactions.

Regarding activity patterns, future studies should assess whether temporal relationships between clouded tiger‐cats and dogs are consistent between sexes, reproductive stages, and under varying levels of light pollution, particularly for the ANSCD. For instance, in a degraded landscape of South Africa, nocturnal activity in caracals ( *Caracal caracal* ) was negatively affected by the Artificial Light at Night (ALAN), leading to active avoidance of illuminated areas and a decreased use of kill sites by this mesopredator (Hickling et al. 2026). Intraspecific variation in wild cat activity can also occur, as individuals in different sexes or reproductive stages (e.g., females rearing offspring or subadults) may differ in their temporal strategies to balance risks avoidance with resource intake (Allen et al. 2024; Hickling et al. 2026; Yen et al. 2019). Finally, we endorse the implementation of hybrid terrestrial and arboreal camera trap surveys to achieve a comprehensive understanding of clouded tiger‐cat spatiotemporal ecology and inform conservation actions (see Dymit et al. 2025; Zwicker et al. 2024).

## 5 Conclusions

Landscape degradation and rampant urban development in the ANSCD have resulted in the geographic isolation of clouded tiger‐cats, which are relegated to use the few remnants of well‐protected forest available in the steepest terrain, especially at night. This situation is concerning, given the vulnerability of the species to inbreeding depression and local extinction, which can be exacerbated by the synergistic and increasing pressures of human modification and dog invasive potential (Yen et al. 2019). We found that dogs used more disturbed and transitional forests while exhibiting diurnal behavior, which can be associated with their dependence on humans. Our results suggest that the nocturnal behavior of clouded tiger‐cats plays an important role in reducing their risk of encounters with dogs, while giving them access to resources and reproductive opportunities. Predators with low‐density populations affected by anthropogenic factors such as landscape modification or poaching are particularly susceptible to dogs, whether dogs invade their habitats alone or in packs (Gompper 2021). The widespread co‐occurrence between clouded tiger‐cats and dogs highlights the need to establish rules and guidelines for dog owners to leash their pets and constrain dog movement both inside and around the ANSCD. In addition, knowledge of dog habitat use and activity in the ANSCD can inform decision makers to select the best hours and places to conduct trap‐neuter‐release, as well as vaccination campaigns with polyvalent schemes (Yen et al. 2019). However, to optimally mitigate the potential contact between dogs and clouded tiger‐cats, these actions need to be implemented in the long term, with sufficient intensity, and in conjunction with wildlife conservation education (Gompper 2021).

## Author Contributions

**J. C. Cepeda‐Duque:** conceptualization (lead), data curation (lead), formal analysis (lead), funding acquisition (equal), investigation (lead), methodology (lead), project administration (lead), writing – original draft (lead), writing – review and editing (lead). **A. M. López‐Barrera:** data curation (equal), investigation (equal), writing – original draft (equal). **E. Arango‐Correa:** data curation (equal), investigation (equal), writing – original draft (equal). **J. F. Hernández‐Fitzgerald:** data curation (equal), investigation (equal), writing – original draft (equal). **V. López‐Velasco:** data curation (equal), investigation (equal), writing – original draft (equal). **L. A. Fox‐Rosales:** conceptualization (equal), data curation (equal), formal analysis (equal), investigation (equal), methodology (equal), validation (equal), writing – original draft (equal), writing – review and editing (equal). **T. G. de Oliveira:** conceptualization (equal), data curation (equal), funding acquisition (equal), methodology (equal), project administration (equal), supervision (equal), validation (equal), writing – original draft (equal), writing – review and editing (equal).

## Funding

This work was supported by Mohamed bin Zayed Species Conservation Fund, #232532256.

## Conflicts of Interest

The authors declare no conflicts of interest.

## Supporting information


**Figure S1:** Pearson correlation matrix of the predictor variables used to determine the habitat use of clouded tiger‐cats and dogs between July 2022 and September 2023 in Alto del Nudo Soil Conservation District. ER; Encounter rate, human_mod; Human Modification Index, FLII; Forest Landscape Integrity Index, dist_pop; Distance to the nearest human settlements, dist_road; Distance to the nearest road; canopy_c; Canopy cover, canopy_h; Canopy height, rodent_ER; small mammal encounter rates (number of detections/sampling effort*100 trap‐nights).
**Figure S2:** Results of the distributional assumptions of the residuals derived from the negative binomial models relating the number of clouded tiger‐cat detections against (a) the Human Modification Index and (b) the Slope predictors, and the number of dog detections against (c) the quadratic canopy cover and (d) the canopy height.
**Figure S3:** Quantile regression of the residuals against predicted values from the top negative binomial models relating the number of clouded tiger‐cat detections against (a) the Human Modification Index and (b) the Slope predictors, and the number of dog detections against (c) the quadratic canopy cover and (d) the canopy height.
**Figure S4:** Residual dispersion test for the negative binomial models relating the number of clouded tiger‐cat detections against (a) the Human Modification Index and (b) the Slope predictors, and the number of dog detections against (c) the quadratic form of canopy cover and (d) the canopy height.
**Figure S5:** Outlier test for the residuals of the negative binomial models relating the number of clouded tiger‐cat detections against (a) the Human Modification Index and (b) the Slope predictors, and the number of dog detections against (c) the quadratic form of the canopy cover and (d) the canopy height.
**Figure S6:** Zero‐inflation tests for the residuals of the negative binomial models relating the number of clouded tiger‐cat detections against (a) the Human Modification Index and (b) the Slope predictors, and the number of dog detections against (c) the quadratic form of canopy cover and (d) the canopy height.
**Figure S7:** Activity curves of clouded tiger‐cats and small mammal prey in the Alto del Nudo Soil Conservation District between July 2022 and September 2023 for the full duration of the study (a), the rainy (b) and dry season (c), sites with high (d) and low (e) dog encounter rates, and human activity in the form of days of working (f) and resting (g). The shaded area is the overlap between the activity curves of both species. The Watson two‐sample (*U* ^2^) test are also provided. The vector image of small mammal prey was obtained from https://www.vecteezy.com/ and of dogs was obtained from https://www.canva.com/.
**Figure S8:** Activity curves of clouded tiger‐cats and dogs in the Alto del Nudo Soil Conservation District between July 2022 and September 2023 for the full duration of the study (a), the rainy (b) and dry season (c), sites with high (d) and low (e) dog encounter rates, and human activity in the form of days of working (f) and resting (g). The shaded area is the overlap between the activity curves of both species. The Watsontwo‐sample (*U* ^2^) test are also provided. The vector image of small mammal prey was obtained from https://www.vecteezy.com/ and of dogs was obtained from https://www.canva.com/.
**Figure S9:** Core activity (time interval of the measured 50% isopleth or the shortest time interval containing 50% of all detections) of clouded tiger‐cats, dogs, and small mammal prey in general, among rainfall seasons, and human activities. The vector image of small mammal prey was obtained from https://www.vecteezy.com/ and of dogs was obtained from https://www.canva.com/.
**Table S1:** Predictors included in the negative binomial models to understand the habitat use of clouded tiger‐cats and dogs for the camera trap survey conducted between July 2022 and September 2023 in the Alto del Nudo Soil Conservation District. The minimum (Min) and maximum (Max) values of each predictor, the corresponding descriptive statistics (mean, median, and standard deviation—SD), and the variance inflation factor (VIF) are provided. In the VIF column, we indicated the resulting VIF value after dropping the colinear predictors (elevation, distance to human settlements) between parentheses.
**Table S2:** Model selection to determine the best structure of the null models to understand the habitat use of clouded tiger‐cats and dogs in the Alto del Nudo Soil Conservation District. We structured the null models to determine the relevance of using linear (LV) or quadratic (QV) variance functions in the negative binomial distribution, including or excluding sampling stations as random effects (RE) and a zero‐inflation parameter (ZI). Df; Degrees of freedom, logLik; Log‐likelihood, AICc; Akaike information criterion for small sample sizes, AIC_∆_; AICc difference, AICw; AIC weight. Since several null models scored an AIC_∆_ < 2, we decided to select the models with simplest structure to avoid overfitting.

## Data Availability

All relevant data and scripts from this study are available in the OSF repository (https://osf.io/9v7c6/).

## References

[ece373770-bib-0001] Agostinelli, C. , and U. Lund . 2022. “R Package Circular: Circular Statistics” Version 0.4–95. https://r‐forge.r‐project.org/projects/circular/.

[ece373770-bib-0002] Allen, M. L. , A. M. Green , A. C. Avrin , and C. C. Wilmers . 2024. “Female Pumas Exhibit Behavioral Plasticity Through Partitioning Temporal Activity at Communication Hubs Based on Life Stage.” Ecological Research 40, no. 1: 56–64. 10.1111/1440-1703.12514.

[ece373770-bib-0003] Allen, M. L. , K. C. Weiss , and A. M. Green . 2025. “Ecological and Anthropogenic Drivers of Red Fox ( *Vulpes vulpes* ) Abundance and Site Use Across the Contiguous USA.” Journal of Biogeography 52, no. 9: e70008. 10.1111/jbi.70008.

[ece373770-bib-0004] Aurich‐Rodriguez, F. , R. P. Piana , R. D. Appleton , and A. C. Burton . 2022. “Threatened Andean Bears Are Negatively Affected by Human Disturbance and Free‐Ranging Cattle in a Protected Area in Northwest Peru.” Mammalian Biology 102, no. 1: 177–187. 10.1007/s42991-021-00217-z.

[ece373770-bib-0005] Avendaño, M. 2019. “Análisis de la Actividad: Circular.” In Fototrampeo en R: Organización y Análisis de Datos, edited by S. Mandujano and L. A. Pérez‐Solano , 167–188. Instituto de Ecología A. C.

[ece373770-bib-0006] Avendaño‐Díaz, M. , C. Delfín‐Alfonso , L. García‐Feria , M. Hidalgo‐Mihart , O. Lagunes‐Merino , and J. E. Morales‐Mávil . 2025. “Foraging Patterns and Spatial Distribution of Synanthropic Mammals and Their Interaction With Dogs.” Revista de Biología Tropical 73, no. 1: e61727. 10.15517/rev.biol.trop..v73i1.61727.

[ece373770-bib-0007] Barrera‐Vargas, J. , C. A. Delgado‐V , and A. Arias‐Alzate . 2023. “Mesocarnivores Activity Patterns in the Northern Colombian Andes.” Therya 14, no. 3: 371–382. 10.12933/THERYA-23-1243.

[ece373770-bib-0008] Bartoń, K. 2025. “MuMIn: Multi‐Model Inference.” Version 1.48.11. https://CRAN.R‐project.org/package=MuMIn.

[ece373770-bib-0009] Bedoya‐Durán, M. J. , O. E. Murillo‐García , and L. C. Branch . 2021. “Factors Outside Privately Protected Areas Determine Mammal Assemblages in a Global Biodiversity Hotspot in the Andes.” Global Ecology and Conservation 32: e01921. 10.1016/j.gecco.2021.e01921.

[ece373770-bib-0010] Beltrami, E. , N. Gálvez , C. Osorio , M. J. Kelly , D. Morales‐Moraga , and C. Bonacic . 2023. “Ravines as Conservation Strongholds for Small Wildcats Under Pressure From Free‐Ranging Dogs and Cats in Mediterranean Landscapes of Chile.” Studies on Neotropical Fauna and Environment 58, no. 1: 138–154. 10.1080/01650521.2021.1933691.

[ece373770-bib-0011] Bianchi, R. , N. Olifiers , L. L. Riski , et al. 2020. “Dog Activity in Protected Areas: Behavioral Effects on Mesocarnivores and the Impacts of a Top Predator.” European Journal of Wildlife Research 66, no. 3: 36. 10.1007/s10344-020-01376-z.

[ece373770-bib-0012] Biswas, S. , K. Ghosh , K. Sarkar , L. Benny , M. Katti , and A. Bhadra . 2024. “A Population‐Level Study Reveals Hidden Patterns in Resting Site Choice of Free‐Ranging Dogs.” Biological Journal of the Linnean Society 143, no. 3: blae095. 10.1093/biolinnean/blae095.

[ece373770-bib-0013] Blanchet, F. G. , K. Cazelles , and D. Gravel . 2020. “Co‐Occurrence Is Not Evidence of Ecological Interactions.” Ecology Letters 23, no. 7: 1050–1063. 10.1111/ele.13525. 32429003

[ece373770-bib-0014] Boller, L. , A. D. Pereira , C. G. Fialek , and S. Bazílio . 2023. “Activity and Overlap Pattern of Medium to Large‐Sized Mammals on Cachoeirinha Municipal Ecological Station in Southern Brazil.” Brazilian Journal of Mammalogy 92: e92202391. 10.32673/bjm.vi92.91.

[ece373770-bib-0015] Bolze, G. J. , F. P. Tirelli , D. Queirolo , and M. J. Ramos Pereira . 2021. “Living on the Edge: Density and Activity Patterns of the Ocelot, *Leopardus pardalis* , in the Austral Limit of the Atlantic Forest.” Studies on Neotropical Fauna and Environment 58, no. 3: 599–612. 10.1080/01650521.2021.2008146.

[ece373770-bib-0017] Botts, R. T. , A. A. Eppert , T. J. Wiegman , et al. 2020. “Does Moonlight Increase Predation Risk for Elusive Mammals in Costa Rica?” Tropical Conservation Science 13: 194008292095240. 10.1177/1940082920952405.

[ece373770-bib-0016] Botts, R. , A. Eppert , T. Wiegman , et al. 2020. “Circadian Activity Patterns of Mammalian Predators and Prey in Costa Rica.” Journal of Mammalogy 101, no. 5: 1313–1331. 10.1093/jmammal/gyaa103. 33343263 PMC7733402

[ece373770-bib-0018] Brooks, M. E. , K. Kristensen , K. J. van Benthem , et al. 2017. “glmmTMB Balances Speed and Flexibility Among Packages for Zero‐Inflated Generalized Linear Mixed Modeling.” R Journal 9, no. 2: 378–400. 10.32614/RJ-2017-066.

[ece373770-bib-0019] Bryce, C. M. 2021. “Dogs as Pets and Pests: Global Patterns of Canine Abundance, Activity, and Health.” Integrative and Comparative Biology 61, no. 1: 154–165. 10.1093/icb/icab046. 33940621

[ece373770-bib-0020] Burnham, K. , and D. Anderson . 2002. “Model Selection and Multimodel Inference: A Practical Information‐Theoretic Approach.” In Bayesian Data Analysis in Ecology Using Linear Models With R, BUGS, and STAN, 2nd ed. Springer.

[ece373770-bib-0021] Carvalho, W. D. , L. M. Rosalino , M. Godoy , et al. 2019. “Temporal Activity of Rural Free‐Ranging Dogs: Implications for the Predator and Prey Species in the Brazilian Atlantic Forest.” NeoBiota 45: 55–74. 10.3897/neobiota.45.30645.

[ece373770-bib-0022] Cepeda‐Duque, J. C. , G. Andrade‐Ponce , A. Montes‐Rojas , et al. 2023. “Assessing Microhabitat, Landscape Features and Intraguild Relationships in the Occupancy of the Enigmatic and Threatened Andean Tiger Cat ( *Leopardus tigrinus pardinoides* ) in the Cloud Forests of Northwestern Colombia.” PLoS One 18, no. 7: e0288247. 10.1371/journal.pone.0288247. 37428730 PMC10332582

[ece373770-bib-0023] Cepeda‐Duque, J. C. , E. Arango‐Correa , G. Andrade‐Ponce , L. Mazariegos , T. Hofmeester , and H. E. Ramírez‐Chaves . 2023. “Expanding the Frontiers of Camera‐Trapping in Colombia: Application of the “Mostela” System to Gain Knowledge on Small Non‐Volant Mammals From an Andean Cloud Forest.” Mammalia 87, no. 5: 419–428. 10.1515/mammalia-2023-0033.

[ece373770-bib-0024] Cepeda‐Duque, J. C. , E. Arango‐Correa , C. Frimodt‐Møller , and D. J. Lizcano . 2024. “Howling Shadows: First Report of Domestic Dog Attacks on Globally Threatened Mountain Tapirs in High Andean Cloud Forests of Colombia.” Neotropical Biology & Conservation 19, no. 1: 25–33. 10.3897/neotropical.19.e117437.

[ece373770-bib-0025] Cepeda‐Duque, J. C. , E. Arango‐Correa , V. López‐Velasco , et al. 2024. “Avoiding the Enemy While Searching for Dinner: Understanding the Temporal Niche of the Threatened Clouded Tiger‐Cat in Protected Cloud Forests of the Middle Cauca, Colombia.” Food Webs 42: e00385. 10.1016/j.fooweb.2024.e00385.

[ece373770-bib-0026] Cepeda‐Duque, J. C. , B. Gómez‐Valencia , S. Alvarez , D. R. Gutiérrez‐Sanabria , and D. J. Lizcano . 2021. “Daily Activity Pattern of Pumas ( *Puma concolor* ) and Their Potential Prey in a Tropical Cloud Forest of Colombia.” Animal Biodiversity and Conservation 44, no. 2: 267–278. 10.32800/abc.2021.44.0267.

[ece373770-bib-0027] Contreras‐Abarca, R. , S. J. Crespin , D. Moreira‐Arce , and J. A. Simonetti . 2022. “Redefining Feral Dogs in Biodiversity Conservation.” Biological Conservation 265: 109434. 10.1016/j.biocon.2021.109434.

[ece373770-bib-0030] de Oliveira, T. G. , L. A. Fox‐Rosales , J. D. Ramírez‐Fernández , et al. 2024. “Ecological Modeling, Biogeography, and Phenotypic Analyses Setting the Tiger Cats' Hyperdimensional Niches Reveal a New Species.” Scientific Reports 14, no. 1: 2395. 10.1038/s41598-024-52379-8. 38287072 PMC10825201

[ece373770-bib-0028] de Oliveira, T. , B. C. Lima , L. Fox‐Rosales , R. S. Pereira , E. Pontes‐Araújo , and A. L. de Sousa . 2020. “A Refined Population and Conservation Assessment of the Elusive and Endangered Northern Tiger Cat ( *Leopardus tigrinus* ) in Its Key Worldwide Conservation Area in Brazil.” Global Ecology and Conservation 22: e00927. 10.1016/j.gecco.2020.e00927.

[ece373770-bib-0029] de Oliveira, T. , and J. Pereira . 2014. “Intraguild Predation and Interspecific Killing as Structuring Forces of Carnivoran Communities in South America.” Journal of Mammalian Evolution 21: 427–436. 10.1007/s10914-013-9251-4.

[ece373770-bib-0031] Dechner, A. , K. M. Flesher , C. Lindell , T. V. de Oliveira , and B. A. Maurer . 2018. “Determining Carnivore Habitat Use in a Rubber/Forest Landscape in Brazil Using Multispecies Occupancy Models.” PLoS One 13, no. 4: e0195311. 10.1371/journal.pone.0195311. 29659594 PMC5901926

[ece373770-bib-0032] Dias, D. d. M. , R. Lima Massara , C. B. de Campos , and H. F. R. Guimarães . 2019. “Human Activities Influence the Occupancy Probability of Mammalian Carnivores in the Brazilian Caatinga.” Biotropica 51, no. 2: 253–265. 10.1111/btp.12628.

[ece373770-bib-0033] Díaz, E. A. , C. Sáenz , G. Segnini , A. Villagómez , R. F. Díaz , and R. Zug . 2021. “Dystocia and Cesarean Section in a Free‐Ranging Ocelot ( *Leopardus pardalis* ) After Traumatic Spinal Cord Injury Resulting From Dog ( *Canis familiaris* ) Attack.” Open Veterinary Journal 11, no. 3: 14. 10.5455/OVJ.2021.v11.i3.14. 34722206 PMC8541711

[ece373770-bib-0034] Díaz, E. A. , C. Sáenz , Y. Vega , et al. 2023. “Dog and Cat‐Related Attacks on Wildlife in the Metropolitan District of Quito, Ecuador: An Integrative Approach to Reduce the Impact.” Ecosystems and People 19, no. 1: 2191735. 10.1080/26395916.2023.2191735.

[ece373770-bib-0035] DiRenzo, G. V. , D. A. W. Miller , and E. H. C. Grant . 2022. “Ignoring Species Availability Biases Occupancy Estimates in Single‐Scale Occupancy Models.” Methods in Ecology and Evolution 13, no. 8: 1790–1804. 10.1111/2041-210X.13881.

[ece373770-bib-0036] Doherty, T. S. , C. R. Dickman , A. S. Glen , et al. 2017. “The Global Impacts of Domestic Dogs on Threatened Vertebrates.” Biological Conservation 210: 56–59. 10.1016/j.biocon.2017.04.007.

[ece373770-bib-0037] dos Santos, C. L. A. , A. P. Silva , S. B. dos Santos , R. Pardini , and C. R. Cassano . 2017. “Dog Invasion in Agroforests: The Importance of Households, Roads and Dog Population Size in the Surroundings.” Perspectives in Ecology and Conservation 15, no. 3: 221–226. 10.1016/j.pecon.2017.08.001.

[ece373770-bib-0038] Dymit, E. M. , J. P. Twining , R. Garcia‐Anleu , J. M. Allen , and T. Levi . 2025. “Niche Partitioning Among Neotropical Felids.” Journal of Animal Ecology 95, no. 1: 115–130. 10.1111/1365-2656.70173. 41276917

[ece373770-bib-0039] Fennell, M. J. , A. T. Ford , T. G. Martin , and A. C. Burton . 2023. “Assessing the Impacts of Recreation on the Spatial and Temporal Activity of Mammals in an Isolated Alpine Protected Area.” Ecology and Evolution 13, no. 11: e10733. 10.1002/ece3.10733. 38034339 PMC10682857

[ece373770-bib-0040] Ferretti, F. , R. Oliveira , M. Rossa , et al. 2023. “Interactions Between Carnivore Species: Limited Spatiotemporal Partitioning Between Apex Predator and Smaller Carnivores in a Mediterranean Protected Area.” Frontiers in Zoology 20, no. 1: 20. 10.1186/s12983-023-00489-w. 37231517 PMC10210480

[ece373770-bib-0041] Frigeri, E. , C. R. Cassano , and R. Pardini . 2014. “Domestic Dog Invasion in an Agroforestry Mosaic in Southern Bahia, Brazil.” Tropical Conservation Science 7, no. 3: 10. 10.1177/194008291400700310.

[ece373770-bib-0042] García, C. B. , G. L. Svensson , C. Bravo , et al. 2021. “Remnants of Native Forests Support Carnivore Diversity in the Vineyard Landscapes of Central Chile.” Oryx 55, no. 2: 227–234. 10.1017/S0030605319000152.

[ece373770-bib-0043] Gerber, B. D. , K. Devarajan , Z. J. Farris , and M. Fidino . 2024. “A Model‐Based Hypothesis Framework to Define and Estimate the Diel Niche via the ‘Diel.Niche’ R Package.” Journal of Animal Ecology 93, no. 2: 132–146. 10.1111/1365-2656.14035. 38213300

[ece373770-bib-0044] Gompper, M. E. 2015. “The Dog‐Human‐Wildlife Interface: Assessing the Scope of the Problem.” In Free‐Ranging Dogs and Wildlife Conservation, edited by M. Gompper , 9–54. Oxford University Press. 10.1093/acprof:osobl/9780199663217.003.0001.

[ece373770-bib-0045] Gompper, M. E. 2021. “Adding Nuance to Our Understanding of Dog–Wildlife Interactions and the Need for Management.” Integrative and Comparative Biology 61, no. 1: 93–102. 10.1093/icb/icab049. 33963410

[ece373770-bib-0046] González‐González, A. , N. Clerici , and B. Quesada . 2022. “A 30 m‐Resolution Land Use‐Land Cover Product for the Colombian Andes and Amazon Using Cloud‐Computing.” International Journal of Applied Earth Observation and Geoinformation 107: 102688. 10.1016/j.jag.2022.102688.

[ece373770-bib-0047] Granados, A. , Z. McDonald , K. McPherson , and D. Stoner . 2024. “Unraveling the Impact of Dog‐Friendly Spaces on Urban–Wildland Pumas and Other Wildlife.” Wildlife Biology: e01290. 10.1002/wlb3.01290.

[ece373770-bib-0048] Granados, A. , C. Sun , J. T. Fisher , et al. 2023. “Mammalian Predator and Prey Responses to Recreation and Land Use Across Multiple Scales Provide Limited Support for the Human Shield Hypothesis.” Ecology and Evolution 13, no. 9: e10464. 37720065 10.1002/ece3.10464 PMC10500421

[ece373770-bib-0049] Grantham, H. S. , A. Duncan , T. D. Evans , et al. 2020. “Anthropogenic Modification of Forests Means Only 40% of Remaining Forests Have High Ecosystem Integrity.” Nature Communications 11, no. 1: 5978. 10.1038/s41467-020-19493-3. PMC7723057 33293507

[ece373770-bib-0050] Greenspoon, L. , E. Krieger , R. Sender , et al. 2023. “The Global Biomass of Wild Mammals.” Proceedings of the National Academy of Sciences of the United States of America 120, no. 10: e2204892120. 10.1073/pnas.2204892120. 36848563 PMC10013851

[ece373770-bib-0122] Griss, S. , S. Riemer , C. Warembourg , et al. 2021. “If They Could Choose: How Would Dogs Spend Their Days? Activity Patterns in Four Populations of Domestic Dogs.” Applied Animal Behaviour Science 243: 105449. 10.1016/j.applanim.2021.105449.

[ece373770-bib-0051] Guzmán‐Aguayo, L. , C. Saucedo , Á. Verdugo‐Martínez , et al. 2025. “Temporal Activity Patterns of Tourists and Pumas *Puma concolor* in Public Areas in the Patagonia National Park, Chile.” Wildlife Biology 2025, no. 5: e01396. 10.1002/wlb3.01396.

[ece373770-bib-0052] Hansen, M. C. , P. V. Potapov , R. Moore , et al. 2013. “High‐Resolution Global Maps of 21st‐Century Forest Cover Change.” Science 342, no. 6160: 850–853. 10.1126/science.1244693. 24233722

[ece373770-bib-0053] Hartig, F. 2024. “DHARMa: Residual Diagnostics for Hierarchical Regression Models.” R Package Version 0.4.7. The Comprehensive R Archive Network. https://github.com/florianhartig/dharma.

[ece373770-bib-0054] Hickling, C. J. , L. E. Serieys , G. R. Leighton , et al. 2026. “Illuminating the Influence of Artificial Light at Night on the Behavior of an Adaptable Carnivore.” Science of the Total Environment 1016: 181469. 10.1016/j.scitotenv.2026.181469. 41610542

[ece373770-bib-0055] Ho, H. C. , T. S. Ding , H. W. Yuan , et al. 2025. “Impacts of Free‐Roaming Dogs on Spatiotemporal Niches of Native Carnivores in Taiwan.” Global Ecology and Conservation 57: e03411. 10.1016/j.gecco.2025.e03411.

[ece373770-bib-0056] Horn, P. E. , M. J. R. Pereira , T. C. Trigo , E. Eizirik , and F. P. Tirelli . 2020. “Margay ( *Leopardus wiedii* ) in the Southernmost Atlantic Forest: Density and Activity Patterns Under Different Levels of Anthropogenic Disturbance.” PLoS One 15, no. 5: e0232013. 10.1371/journal.pone.0232013. 32374736 PMC7202647

[ece373770-bib-0057] Hughes, J. , and D. W. Macdonald . 2013. “A Review of the Interactions Between Free‐Roaming Domestic Dogs and Wildlife.” Biological Conservation 157: 341–351. 10.1016/j.biocon.2012.07.005.

[ece373770-bib-0058] Jiménez, G. , N. López‐Cepeda , A. P. Delgado , A. M. Guevara , and L. Lozano . 2017. “Monitoring Program for Mammals in a Protected Area of Colombia.” Universitas Scientiarum 22, no. 1: 9–29. 10.11144/Javeriana.SC22-1.mpfm.

[ece373770-bib-0059] Justa, P. , and S. Lyngdoh . 2023. “Understanding Carnivore Interactions in a Cold Arid Trans‐Himalayan Landscape: What Drives Co‐Existence Patterns Within Predator Guild Along Varying Resource Gradients?” Ecology and Evolution 13, no. 5: e10040. 10.1002/ece3.10040. 37181213 PMC10173057

[ece373770-bib-0060] Landler, L. , G. D. Ruxton , and E. P. Malkemper . 2019. “The Hermans‐Rasson Test as a Powerful Alternative to the Rayleigh Test for Circular Statistics in Biology.” BMC Ecology 19, no. 1: 30. 10.1186/s12898-019-0246-8. 31391040 PMC6686250

[ece373770-bib-0061] Lazzeri, L. , P. Fazzi , M. Lucchesi , et al. 2022. “The Rhythm of the Night: Patterns of Activity of the European Wildcat in the Italian Peninsula.” Mammalian Biology 102, no. 5–6: 1769–1782. 10.1007/s42991-022-00276-w.

[ece373770-bib-0062] Lescroart, J. , A. Bonilla‐Sánchez , C. Napolitano , et al. 2023. “Extensive Phylogenomic Discordance and the Complex Evolutionary History of the Neotropical Cat Genus *Leopardus* .” Molecular Biology and Evolution 40, no. 12: msad255. 10.1093/molbev/msad255. 37987559 PMC10701098

[ece373770-bib-0063] Londoño, G. A. , C. A. Saavedra‐R , D. Osorio , and J. Martínez . 2004. “Notas Sobre la Anidación del Tororoi Bigotudo ( *Grallaria alleni* ) en la Cordillera Central de Colombia.” Ornitología Colombiana 2: 19–24. 10.59517/oc.e29.

[ece373770-bib-0064] Lonsinger, R. C. 2022. “Co‐Occurrence Models Fail to Infer Underlying Patterns of Avoidance and Aggregation When Closure Is Violated.” Ecology and Evolution 12, no. 7: e9104. 10.1002/ece3.910. 35845361 PMC9273567

[ece373770-bib-0065] Maher, E. K. , M. P. Ward , and V. J. Brookes . 2019. “Investigation of the Temporal Roaming Behaviour of Free‐Roaming Domestic Dogs in Indigenous Communities in Northern Australia to Inform Rabies Incursion Preparedness.” Scientific Reports 9, no. 1: 14893. 10.1038/s41598-019-51447-8. 31624301 PMC6797733

[ece373770-bib-0066] Malhotra, R. , J. E. Jiménez , and N. Harris . 2021. “Patch Characteristics and Domestic Dogs Differentially Affect Carnivore Space Use in Fragmented Landscapes in Southern Chile.” Diversity and Distributions 27, no. 11: 2190–2203. 10.1111/ddi.13391.

[ece373770-bib-0067] Mandujano, S. 2024. “Índice de Abundancia Relativa y Tasa de Encuentro Con Trampas Cámara.” Mammalogy Notes 10, no. 1: 389. 10.47603/mano.v10n1.389.

[ece373770-bib-0068] Manzo, I. A. , R. Gonçalves da Silva , and R. C. Bianchi . 2025. “The Influence of Domestic Dogs on the Spatial and Temporal Distribution of Tayra.” Wildlife Biology 2025, no. 2: 1298. 10.1002/wlb3.01298.

[ece373770-bib-0069] Marinho, P. H. , D. Bezerra , M. Antongiovanni , C. R. Fonseca , and E. M. Venticinque . 2018. “Activity Patterns of the Threatened Northern Tiger Cat *Leopardus tigrinus* and Its Potential Prey in a Brazilian Dry Tropical Forest.” Mammalian Biology 89: 30–36. 10.1016/j.mambio.2017.12.004.

[ece373770-bib-0070] Marneweck, C. J. , B. L. Allen , A. R. Butler , et al. 2022. “Middle‐Out Ecology: Small Carnivores as Sentinels of Global Change.” Mammal Review 52, no. 4: 471–479. 10.1111/mam.12300.

[ece373770-bib-0071] Massara, R. , A. M. d. O. Paschoal , L. F. Bailey , P. Doherty , A. Hirsch , and G. A. Chiarello . 2018. “Factors Influencing Ocelot Occupancy in Brazilian Atlantic Forest Reserves.” Biotropica 50, no. 1: 12481. 10.1111/btp.12481.

[ece373770-bib-0073] Mooring, M. S. , A. A. Eppert , and R. T. Botts . 2020. “Natural Selection of Melanism in Costa Rican Jaguar and Oncilla: A Test of Gloger's Rule and the Temporal Segregation Hypothesis.” Tropical Conservation Science 13: 364. 10.1177/1940082920910364.

[ece373770-bib-0074] Moreira‐Arce, D. , P. M. Vergara , and S. Boutin . 2015. “Diurnal Human Activity and Introduced Species Affect Occurrence of Carnivores in a Human‐Dominated Landscape.” PLoS One 10, no. 9: e0137854. 10.1371/journal.pone.0137854. 26368395 PMC4569270

[ece373770-bib-0075] O'Brien, T. G. 2011. “Abundance, Density and Relative Abundance: A Conceptual Framework.” In Camera Traps in Animal Ecology: Methods and Analyses, edited by A. F. O'Connell , J. D. Nichols , and U. Karanth , 71–96. Springer Japan. 10.1007/978-4-431-99495-4_6.

[ece373770-bib-0076] O'Brien, T. G. , M. F. Kinnaird , and H. T. Wibisono . 2003. “Crouching Tigers, Hidden Prey: Sumatran Tiger and Prey Populations in a Tropical Forest Landscape.” Animal Conservation 6, no. 2: 131–139. 10.1017/S1367943003003172.

[ece373770-bib-0077] Oksanen, J. , P. Legendre , B. O'Hara , et al. 2020. “Vegan: Community Ecology Package.” R Package Version 2.5–7. Community Ecology Package 10 (10).

[ece373770-bib-0078] Oliveira‐Santos, L. G. R. , C. A. Zucco , and C. Agostinelli . 2013. “Using Conditional Circular Kernel Density Functions to Test Hypotheses on Animal Circadian Activity.” Animal Behaviour 85, no. 1: 269–280. 10.1016/j.anbehav.2012.09.033.

[ece373770-bib-0079] Orduña‐Villaseñor, M. , D. Valenzuela‐Galván , and J. E. Schondube . 2023. “Tus Mejores Amigos Pueden Ser Tus Peores Enemigos: Impactos de los Gatos y Perros Domésticos en Países Megadiversos.” Revista Mexicana de Biodiversidad 94: e944850. 10.22201/ib.20078706e.2023.94.4850.

[ece373770-bib-0080] Paschoal, A. M. O. , R. L. Massara , L. Bailey , et al. 2018. “Anthropogenic Disturbances Drive Domestic Dog Use of Atlantic Forest Protected Areas.” Tropical Conservation Science 11: 1940082918789833. 10.1177/1940082918789833.

[ece373770-bib-0081] Paschoal, A. M. O. , R. L. Massara , J. L. Santos , and A. G. Chiarello . 2012. “Is the Domestic Dog Becoming an Abundant Species in the Atlantic Forest? A Study Case in Southeastern Brazil.” Mammalia 76, no. 1: 67–76. 10.1515/mammalia-2012-0501.

[ece373770-bib-0082] Payán, E. , and T. G., de Oliveira . 2016. “ *Leopardus tigrinus* .” IUCN Red List. 10.2305/IUCN.UK.2016-2.RLTS.T54012637A50653881.en.

[ece373770-bib-0083] Pereda Sánchez, A. , C. Calvo‐Mac , W. E. Flores Miranda , M. De la Puente‐León , and I. G. Cerna‐Chihuala . 2023. “Patrones de Actividad y Superposición Temporal Entre Carnívoros Nativos y Exóticos en Remanentes Sureños de Bosque Seco Tumbesino en Perú.” Ecología Austral 33, no. 2: 507–515. 10.25260/ea.23.33.2.0.1985.

[ece373770-bib-0084] Pereira, J. A. , N. G. Fracassi , V. Rago , et al. 2010. “Causes of Mortality in a Geoffroy's Cat Population‐a Long‐Term Survey Using Diverse Recording Methods.” European Journal of Wildlife Research 56, no. 6: 939–942. 10.1007/s10344-010-0423-8.

[ece373770-bib-0085] Pinna, A. C. , and L. Magallanes . 2020. “Nuevo Registro de Yaguarundí *Herpailurus yagouaroundi* (Lacépède, 1809) (Mammalia: Carnivora: Felidae) en el Extremo Sur de Brasil.” Notas Sobre Mamíferos Sudamericanos 02, no. 1: 1–5. 10.31687/saremnms.20.0.28.

[ece373770-bib-0086] Potapov, P. , X. Li , A. Hernandez‐Serna , et al. 2021. “Mapping Global Forest Canopy Height Through Integration of GEDI and Landsat Data.” Remote Sensing of Environment 253: 112165. 10.1016/j.rse.2020.112165.

[ece373770-bib-0087] R Core Team . 2024. “R: A Language and Environment for Statistical Computing.” R Foundation for Statistical Computing: Vienna, Austria. https://www.R‐project.org.

[ece373770-bib-0088] Ramírez‐Fernández, J. D. , L. A. Fox‐Rosales , M. S. Mooring , et al. 2024. “Distribution and Habitat Use Patterns of the Endangered Central American Clouded Oncilla (*Leopardus pardinoides oncilla*) in Costa Rica.” PLoS One 19, no. 9: e0310562. 10.1371/journal.pone.0310562. 39288115 PMC11407673

[ece373770-bib-0089] Reasoner, E. , L. Marker , S. Verschueren , W. D. Briers‐Louw , M. Mbidzo , and B. Cristescu . 2024. “Relative Abundance of a Mesocarnivore in a Human‐Dominated, Semi‐Arid Rangeland in Namibia.” Frontiers in Ecology and Evolution 12: 1333162. 10.3389/fevo.2024.1333162.

[ece373770-bib-0090] Reilly, M. L. , M. W. Tobler , D. L. Sonderegger , and P. Beier . 2017. “Spatial and Temporal Response of Wildlife to Recreational Activities in the San Francisco Bay Ecoregion.” Biological Conservation 207: 117–126. 10.1016/j.biocon.2016.11.003.

[ece373770-bib-0091] Ribeiro, F. S. , E. Nichols , R. G. Morato , J. P. Metzger , and R. Pardini . 2019. “Disturbance or Propagule Pressure? Unravelling the Drivers and Mapping the Intensity of Invasion of Free‐Ranging Dogs Across the Atlantic Forest Hotspot.” Diversity and Distributions 25, no. 2: 191–204. 10.1111/ddi.12845.

[ece373770-bib-0092] Ridout, M. , and M. Linkie . 2009. “Estimating Overlap of Daily Activity Patterns From Camera Trap Data.” Journal of Agricultural, Biological, and Environmental Statistics 14, no. 3: 322–337.

[ece373770-bib-0093] Ritchie, E. G. , C. R. Dickman , M. Letnic , and A. T. Vanak . 2015. “Dogs as Predators and Trophic Regulators.” In Free‐Ranging Dogs and Wildlife Conservation, edited by M. Gompper , 55–78. Oxford University Press. 10.1093/acprof:osobl/9780199663217.003.0002.

[ece373770-bib-0094] Rodríguez, N. , D. Armenteras , and J. Retana . 2013. “Effectiveness of Protected Areas in the Colombian Andes: Deforestation, Fire and Land‐Use Changes.” Regional Environmental Change 13, no. 2: 423–435. 10.1007/s10113-012-0356-8.

[ece373770-bib-0095] Rodríguez‐León, D. S. , and H. F. López‐Arévalo . 2019. “Relative Abundance Variation of Dogs Over a Human Presence Gradient Inside Two Private Nature Reserves (Tabio, Cundinamarca).” Acta Biologica Colombiana 24, no. 2: 379–390. 10.15446/abc.v24n2.70608.

[ece373770-bib-0097] Santon, M. , F. Korner‐Nievergelt , N. K. Michiels , and N. Anthes . 2023. “A Versatile Workflow for Linear Modelling in R.” Frontiers in Ecology and Evolution 11: 1065273. 10.3389/fevo.2023.1065273.

[ece373770-bib-0098] Silva‐Rodríguez, E. A. , and G. R. Ortega‐Solís . 2007. “Human Attitudes Toward Wild Felids in a Human‐Dominated Landscape of Southern Chile.” Cat News. 46.

[ece373770-bib-0099] Smith, K. 2025. “An Empirical Assessment of the Role of Independence Filters in Temporal Activity Analyses Using Camera Trapping Data.” Behavioral Ecology and Sociobiology 79, no. 1: 2. 10.1007/s00265-024-03544-6.

[ece373770-bib-0100] Soultan, A. , O. Attum , and W. Lahue . 2021. “The Relationship Between Landscape Features and Domestic Species on the Occupancy of Native Mammals in Urban Forests.” Urban Ecosystems 24, no. 6: 1117–1128. 10.1098/rstb.2011.0115.

[ece373770-bib-0101] Sousa‐Guedes, D. , A. M. Barbosa , S. Arenas‐Castro , J. C. Campos , and N. Sillero . 2026. “When Good Fit Goes Bad: Identifying and Minimizing Overfitting in Ecological Niche Models.” Journal of Biogeography 53, no. 2: e70165. 10.1111/jbi.70165.

[ece373770-bib-0102] Stobo‐Wilson, A. M. , D. Stokeld , L. D. Einoder , et al. 2020. “Habitat Structural Complexity Explains Patterns of Feral Cat and Dingo Occurrence in Monsoonal Australia.” Diversity and Distributions 26, no. 7: 832–842. 10.1111/ddi.13065.

[ece373770-bib-0103] Sutherland, C. , D. Hare , P. J. Johnson , D. W. Linden , R. A. Montgomery , and E. Droge . 2023. “Practical Advice on Variable Selection and Reporting Using Akaike Information Criterion.” Proceedings of the Royal Society B: Biological Sciences 290, no. 2007: 20231261. 10.1098/rspb.2023.1261. PMC10523071 37752836

[ece373770-bib-0104] Theobald, D. M. , J. R. Oakleaf , G. Moncrieff , M. Voigt , J. Kiesecker , and C. M. Kennedy . 2025. “Global Extent and Change in Human Modification of Terrestrial Ecosystems From 1990 to 2022.” Scientific Data 12, no. 1: 606. 10.1038/s41597-025-04892-2. 40210896 PMC11985953

[ece373770-bib-0105] Thieurmel, B. , and A. Elmarhraoui . 2019. “Suncalc: Compute Sun Position, Sunlight Phases, Moon Position and Lunar Phase.”

[ece373770-bib-0106] Twining, J. P. , B. C. Augustine , J. A. Royle , and A. K. Fuller . 2024. “Abundance‐Mediated Species Interactions.” Ecology 106, no. 1: e4468. 10.1002/ecy.4468. 39633243 PMC11725697

[ece373770-bib-0107] Twining, J. P. , and K. F. Kellner . 2025. “Can Hierarchical Modelling of Co‐Occurrence Data Provide Accurate Inference Into Species Interactions?” Methods in Ecology and Evolution 17, no. 2: 456–479. 10.1111/2041-210x.70210.

[ece373770-bib-0108] Van Scoyoc, A. , J. A. Smith , K. M. Gaynor , K. Barker , and J. S. Brashares . 2023. “The Influence of Human Activity on Predator–Prey Spatiotemporal Overlap.” Journal of Animal Ecology 92, no. 6: 1124–1134. 10.1111/1365-2656.13892. 36710603

[ece373770-bib-0109] Vanak, A. T. , C. R. Dickman , E. A. Silva‐Rodriguez , J. R. A. Butler , and E. G. Ritchie . 2015. “Top‐Dogs and Under‐Dogs: Competition Between Dogs and Sympatric Carnivores.” In Free‐Ranging Dogs and Wildlife Conservation, edited by M. Gompper , 69–93. Oxford University Press. 10.1093/acprof:osobl/9780199663217.003.0003.

[ece373770-bib-0110] Watts, S. M. , T. M. McCarthy , and T. Namgail . 2019. “Modelling Potential Habitat for Snow Leopards ( *Panthera uncia* ) in Ladakh, India.” PLoS One 14, no. 1: e0211509. 10.1371/journal.pone.0211509. 30695083 PMC6350993

[ece373770-bib-0111] Weng, Y. , W. McShea , Y. Diao , et al. 2022. “The Incursion of Free‐Ranging Dogs Into Protected Areas: A Spatio‐Temporal Analysis in a Network of Giant Panda Reserves.” Biological Conservation 265: 109423. 10.1016/j.biocon.2021.109423.

[ece373770-bib-0112] Weston, M. A. , and T. Stankowich . 2015. “Dogs as Agents of Disturbance.” In Free‐Ranging Dogs and Wildlife Conservation, edited by M. Gompper , 94–116. Oxford University Press. 10.1093/acprof:osobl/9780199663217.003.0004.

[ece373770-bib-0113] Yen, S. C. , Y. T. Ju , P. J. L. Shaner , and H. L. Chen . 2019. “Spatial and Temporal Relationship Between Native Mammals and Free‐Roaming Dogs in a Protected Area Surrounded by a Metropolis.” Scientific Reports 9, no. 1: 8161. 10.1038/s41598-019-44474-y. 31160614 PMC6546781

[ece373770-bib-0114] Young, J. K. , K. A. Olson , R. P. Reading , S. Amgalanbaatar , and J. Berger . 2011. “Is Wildlife Going to the Dogs? Impacts of Feral and Free‐Roaming Dogs on Wildlife Populations.” Bioscience 61, no. 2: 125–132. 10.1525/bio.2011.61.2.7.

[ece373770-bib-0115] Zanin, M. , C. L. Bergamaschi , J. R. Ferreira , S. L. Mendes , and D. O. Moreira . 2019. “Dog Days Are Just Starting: The Ecology Invasion of Free‐Ranging Dogs ( *Canis familiaris* ) in a Protected Area of the Atlantic Forest.” European Journal of Wildlife Research 65, no. 5: 5. 10.1007/s10344-019-1303-5.

[ece373770-bib-0116] Zanón Martínez, J. I. , J. Seoane , M. J. Kelly , J. H. Sarasola , and A. Travaini . 2022. “Assessing Carnivore Spatial Co‐Occurrence and Temporal Overlap in the Face of Human Interference in a Semiarid Forest.” Ecological Applications 32, no. 1: e02482. 10.1002/eap.2482. 34674337

[ece373770-bib-0117] Zapata‐Ríos, G. , and L. C. Branch . 2016. “Altered Activity Patterns and Reduced Abundance of Native Mammals in Sites With Feral Dogs in the High Andes.” Biological Conservation 193: 9–16. 10.1016/j.biocon.2015.10.016.

[ece373770-bib-0118] Zapata‐Ríos, G. , and L. C. Branch . 2018. “Mammalian Carnivore Occupancy Is Inversely Related to Presence of Domestic Dogs in the High Andes of Ecuador.” PLoS One 13, no. 2: e0192346. 10.1371/journal.pone.0192346. 29489855 PMC5830290

[ece373770-bib-0119] Zarco‐González, Z. , R. Carrera‐Treviño , Á. Balbuena‐Serrano , J. L. García‐Rivas , and O. Monroy‐Vilchis . 2025. “Spatial Ecology of Black Bears ( *Ursus americanus* ) in Northeastern Mexico: Home Ranges and Habitat Use.” Environmental Monitoring and Assessment 198, no. 1: 14. 10.1007/s10661-025-14853-2. 41350488

[ece373770-bib-0120] Zuur, A. F. , E. N. Ieno , and C. S. Elphick . 2010. “A Protocol for Data Exploration to Avoid Common Statistical Problems.” Methods in Ecology and Evolution 1, no. 1: 3–14. 10.1111/j.2041-210X.2009.00001.x.

[ece373770-bib-0121] Zwicker, S. , C. Sanchez‐Latorre , and C. Lukasser . 2024. “Increasing Detections of the Margay: Occupancy, Density, and Activity Patterns in Madre de Dios, Peru.” Frontiers in Ecology and Evolution 12: 1500202. 10.3389/fevo.2024.1500202.

