# Supplementary material for: Natives Against Invaders: Shared Use of Space and Temporal Segregation of Clouded Tiger‐Cats (Leopardus pardinoides) and Domestic Dogs (Canis familiaris) in an Isolated Protected Area
Source: Ecol Evol. 2026 Jun 3;16(6):e73770. doi: 10.1002/ece3.73770 (PMC13240262; doi:10.1002/ece3.73770)
Supplement: Supplementary file 1 — Figure S1: Pearson correlation matrix of the predictor variables used to determine the habitat use of clouded tiger‐cats and dogs between July 2022 and September 2023 in Alto del Nudo Soil Conservation District. ER; Encounter rate, human_mod; Human Modification Index, FLII; Forest Landscape Integrity Index, dist_pop; Distance to the nearest human settlements, dist_road; Distance to the nearest road; canopy_c; Canopy cover, canopy_h; Canopy height, rodent_ER; small mammal encounter rates (number of detections/sampling effort*100 trap‐nights). Figure S2: Results of the distributional assumptions of the residuals derived from the negative binomial models relating the number of clouded tiger‐cat detections against (a) the Human Modification Index and (b) the Slope predictors, and the number of dog detections against (c) the quadratic canopy cover and (d) the canopy height. Figure S3: Quantile regression of the residuals against predicted values from the top negative binomial models relating the number of clouded tiger‐cat detections against (a) the Human Modification Index and (b) the Slope predictors, and the number of dog detections against (c) the quadratic canopy cover and (d) the canopy height. Figure S4: Residual dispersion test for the negative binomial models relating the number of clouded tiger‐cat detections against (a) the Human Modification Index and (b) the Slope predictors, and the number of dog detections against (c) the quadratic form of canopy cover and (d) the canopy height. Figure S5: Outlier test for the residuals of the negative binomial models relating the number of clouded tiger‐cat detections against (a) the Human Modification Index and (b) the Slope predictors, and the number of dog detections against (c) the quadratic form of the canopy cover and (d) the canopy height. Figure S6: Zero‐inflation tests for the residuals of the negative binomial models relating the number of clouded tiger‐cat detections against (a) the Human Modification Index and [file ECE3-16-e73770-s001.docx]

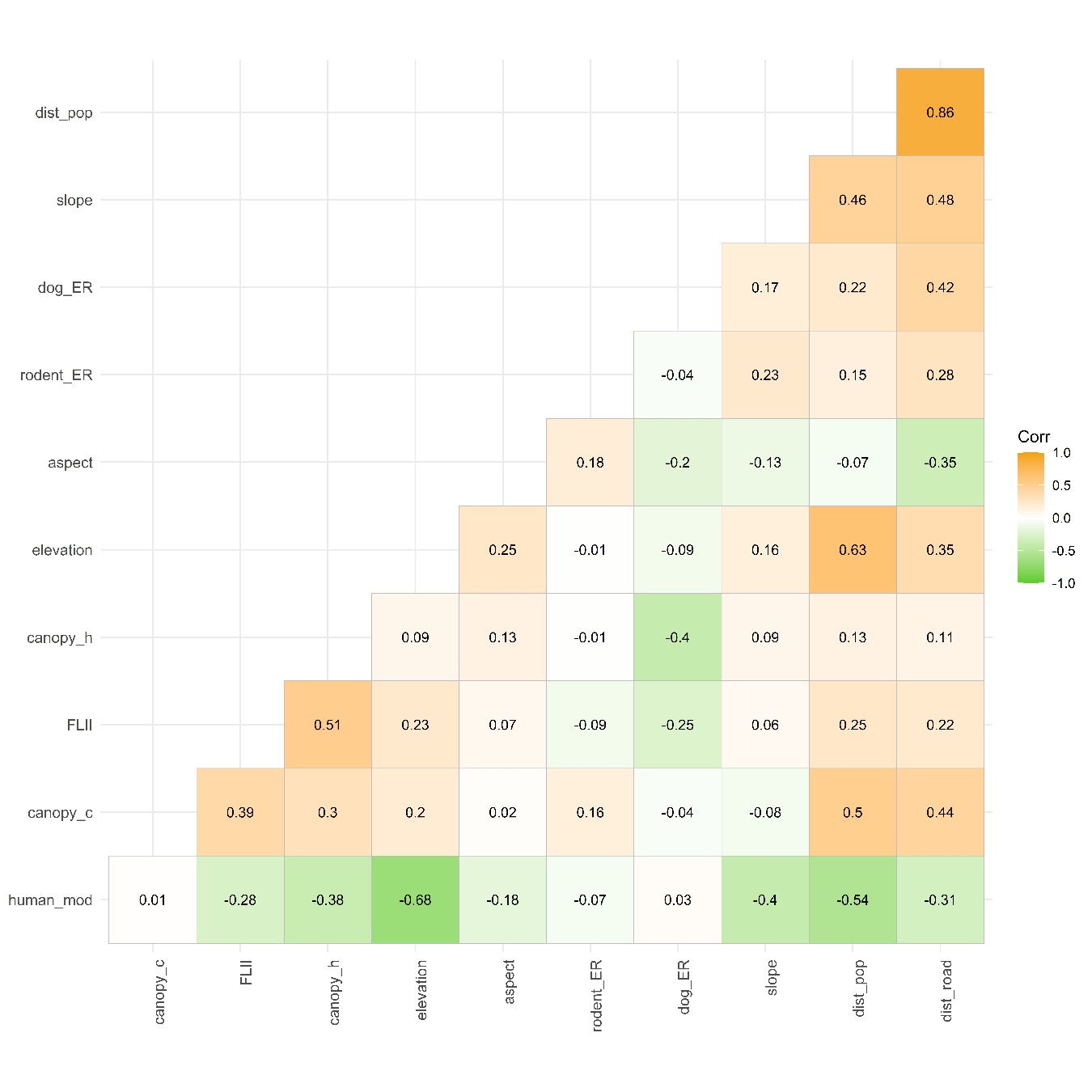
**Figure S1.** Pearson correlation matrix of the predictor variables used to determine the habitat use of clouded tiger-cats and dogs between September 2022 and September 2023 in Alto del Nudo Soil Conservation District. ER; Encounter rate, human_mod; Human Modification Index, FLII; Forest Landscape Integrity Index, dist_pop; Distance to the nearest human settlements, dist_road; Distance to the nearest road; canopy_c; Canopy cover, canopy_h; Canopy height, rodent_ER; small mammal encounter rates (number of detections/sampling effort*100 trap-nights).


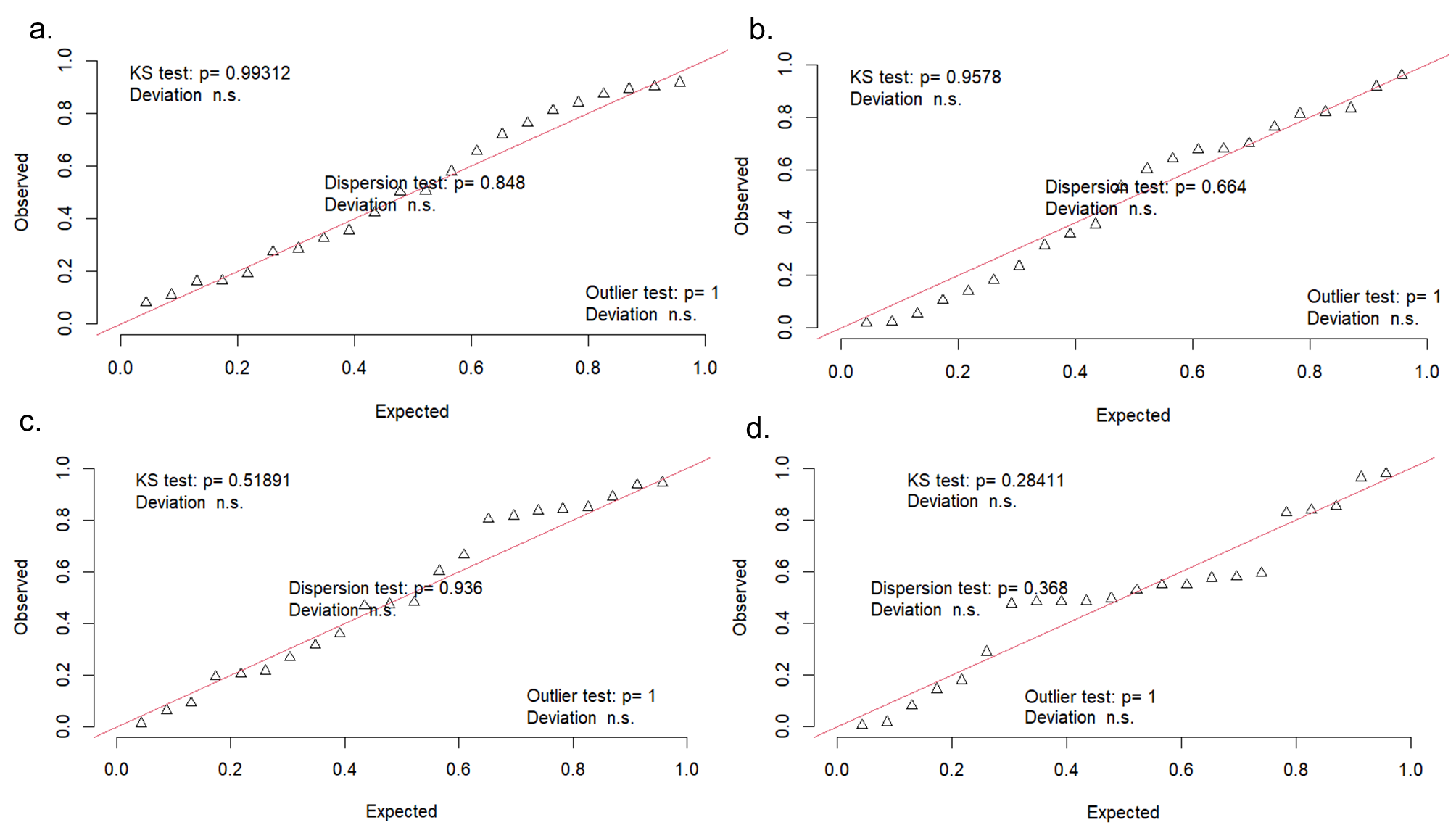


**Figure S2.** Results of the distributional assumptions of the residuals derived from the negative binomial models relating the number of clouded tiger-cat detections against (a) the Human Modification Index and (b) the Slope predictors, and the number of dog detections against (c) the quadratic canopy cover and (d) the canopy height.

**
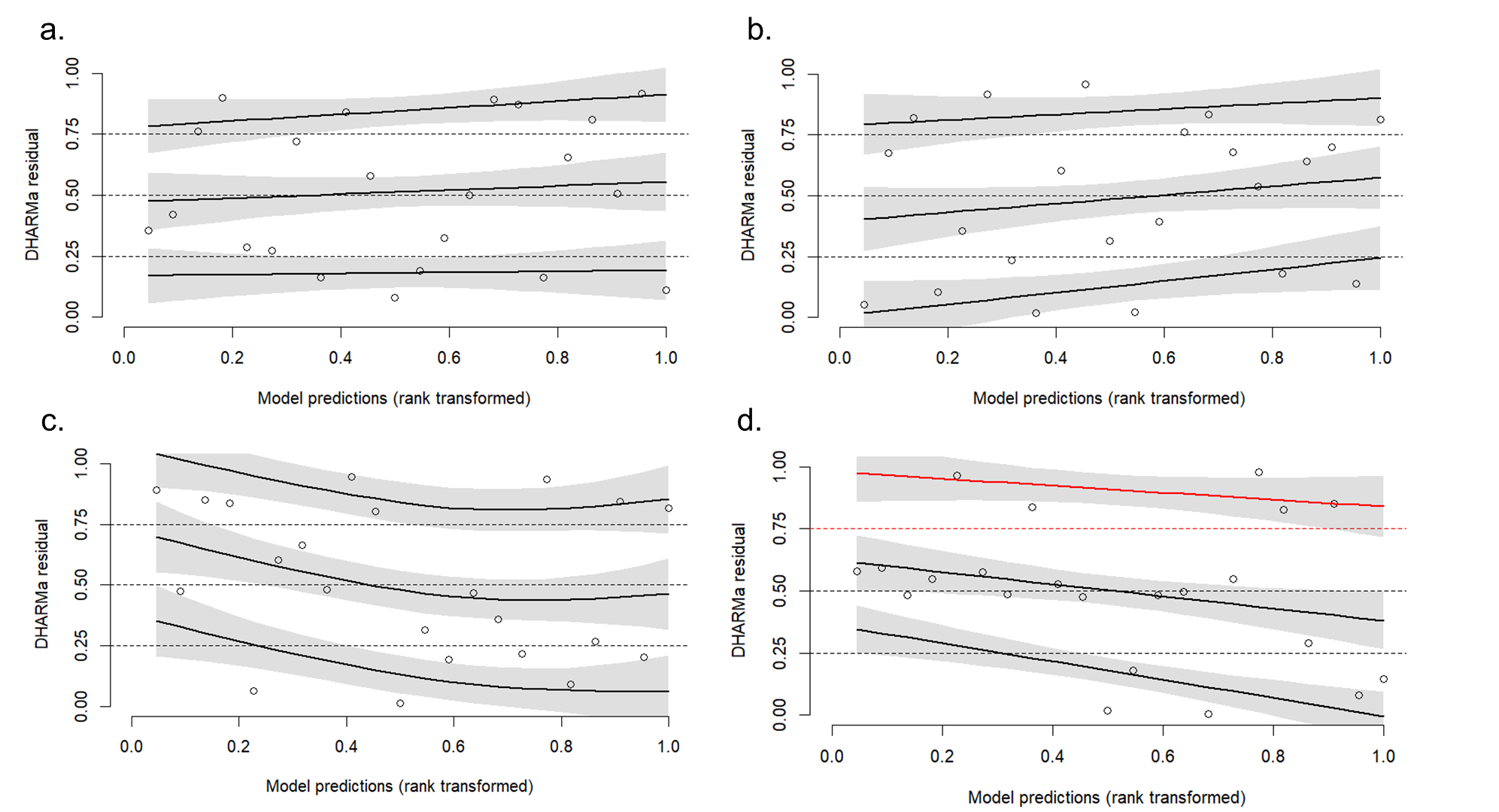
**

**Figure S3.** Quantile regression of the residuals against predicted values from the top negative binomial models relating the number of clouded tiger-cat detections against (a) the Human Modification Index and (b) the Slope predictors, and the number of dog detections against (c) the quadratic canopy cover and (d) the canopy height.

**
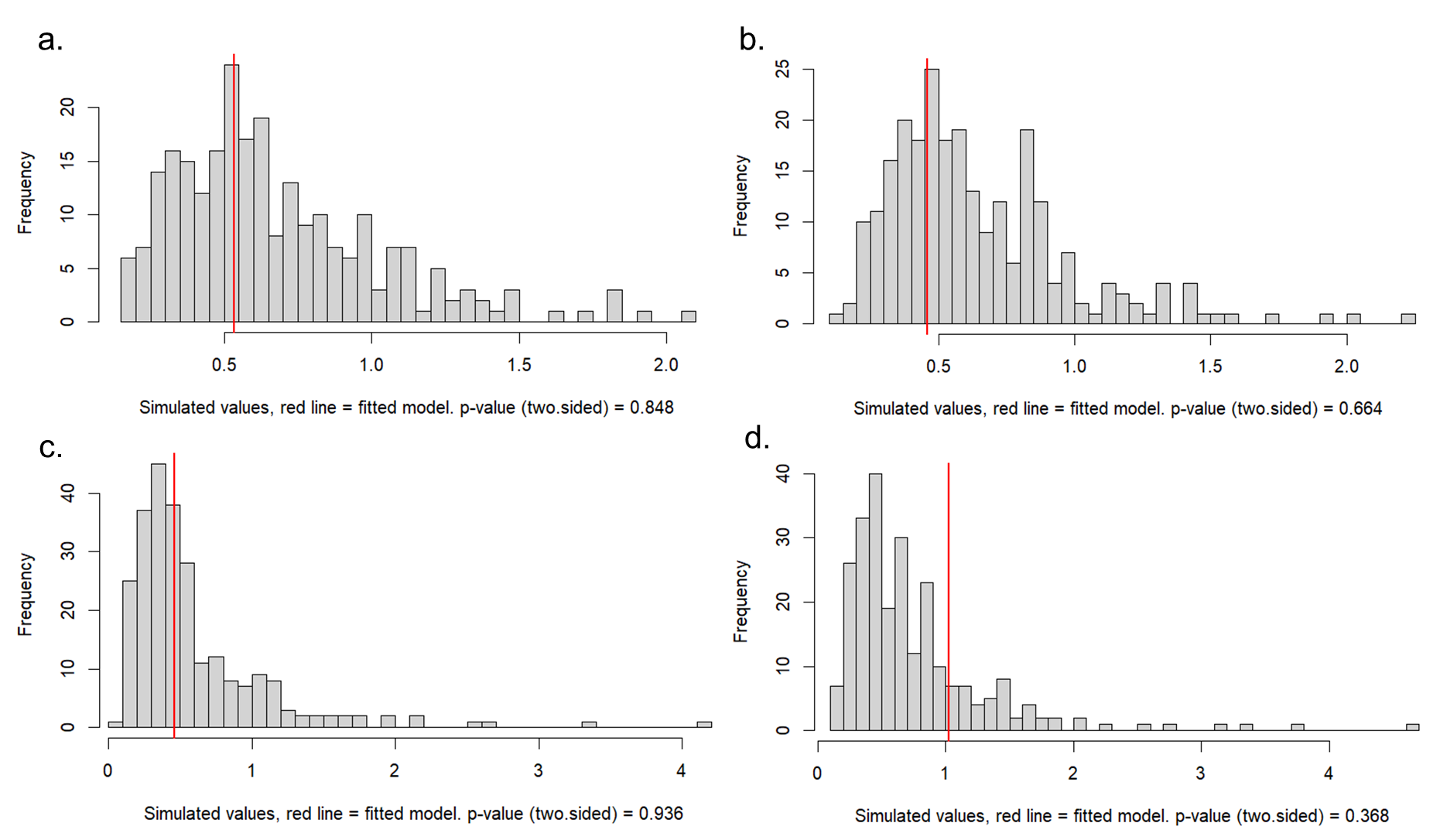
**

**Figure S4.** Residual dispersion test for the negative binomial models relating the number of clouded tiger-cat detections against (a) the Human Modification Index and (b) the Slope predictors, and the number of dog detections against (c) the quadratic form of canopy cover and (d) the canopy height.


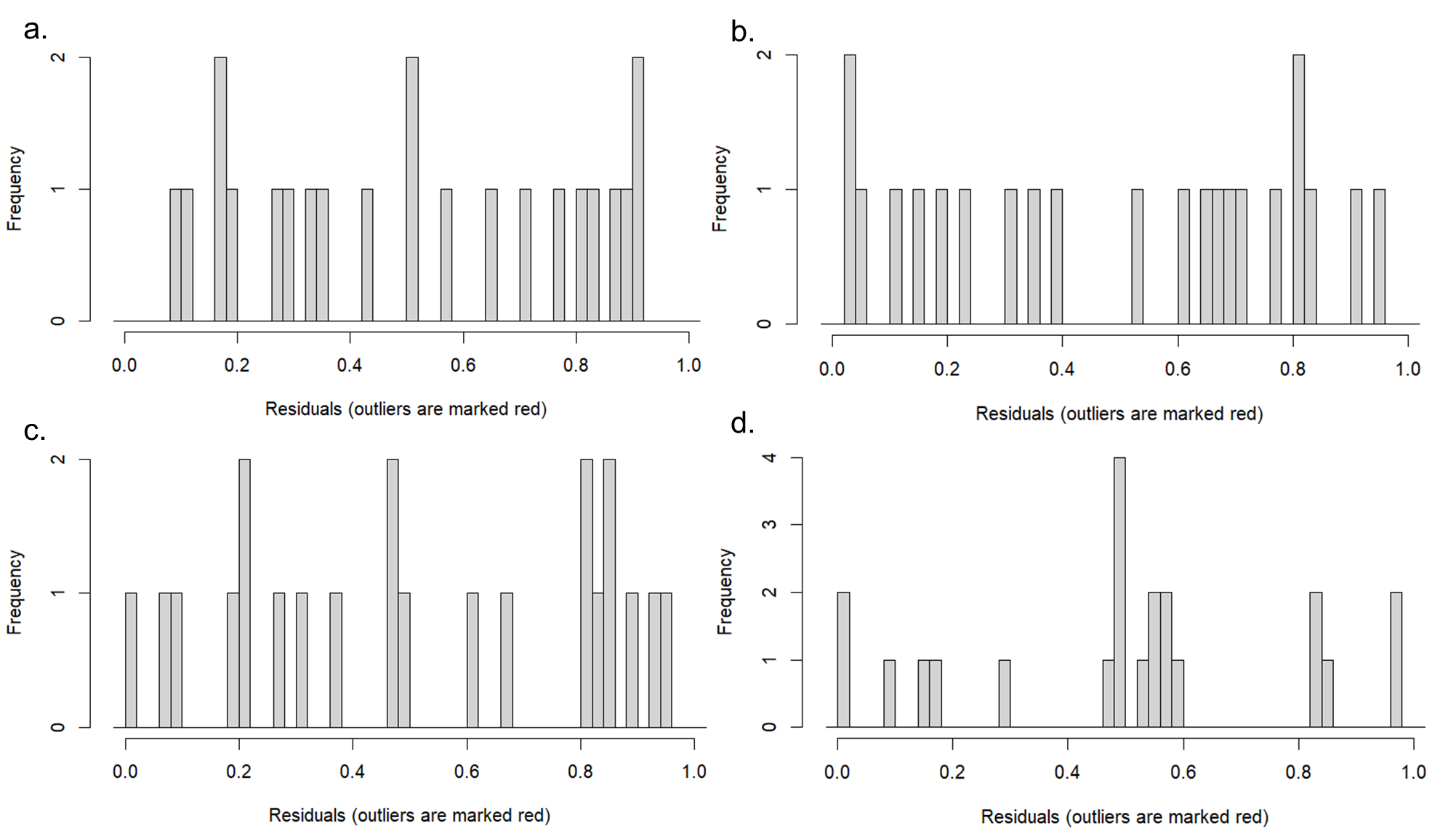


**Figure S5.** Outlier test for the residuals of the negative binomial models relating the number of clouded tiger-cat detections against (a) the Human Modification Index and (b) the Slope predictors, and the number of dog detections against (c) the quadratic form of the canopy cover and (d) the canopy height.

**
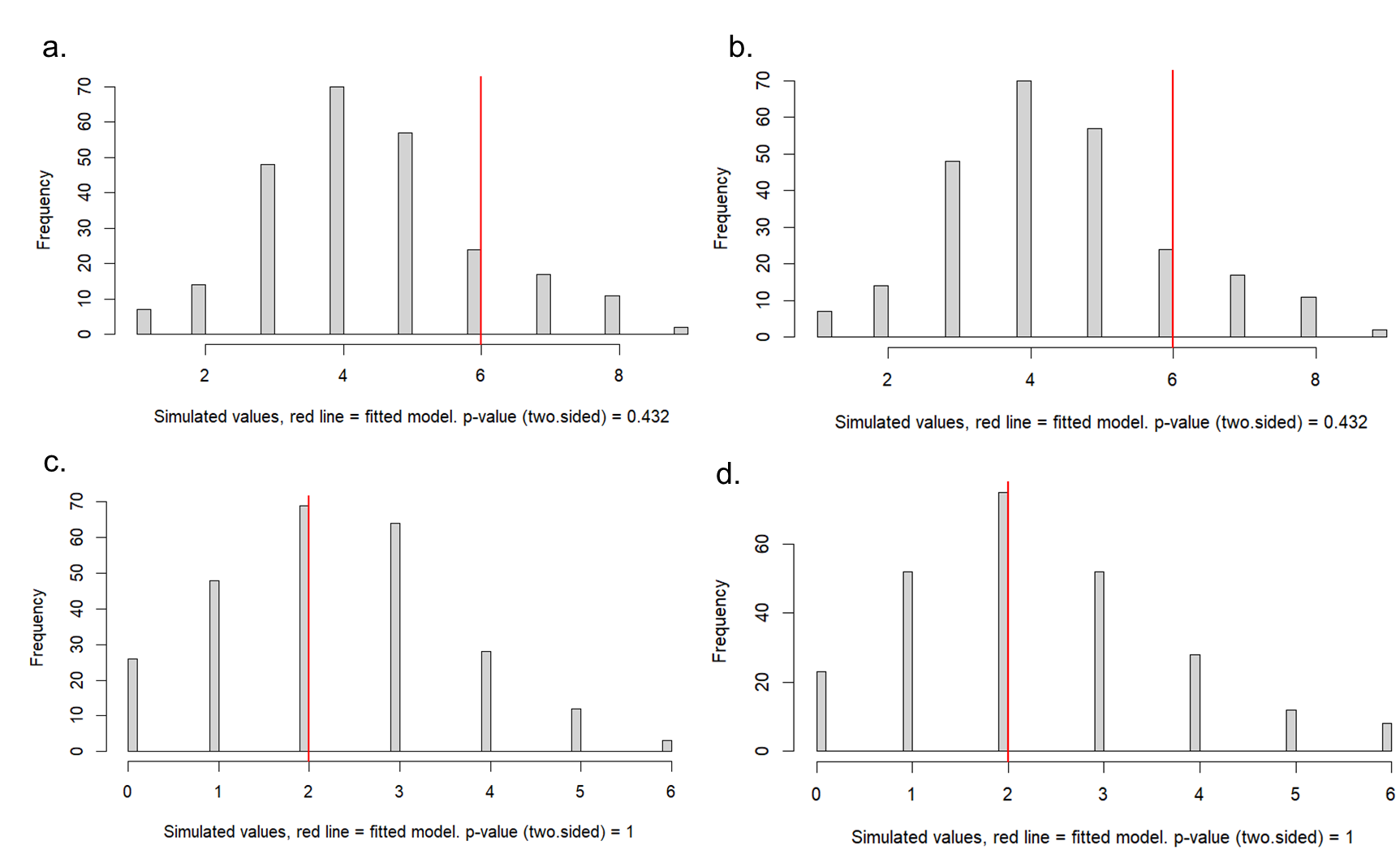
**

**Figure S6.** Zero-inflation tests for the residuals of the negative binomial models relating the number of clouded tiger-cat detections against (a) the Human Modification Index and (b) the Slope predictors, and the number of dog detections against (c) the quadratic form of canopy cover and (d) the canopy height.


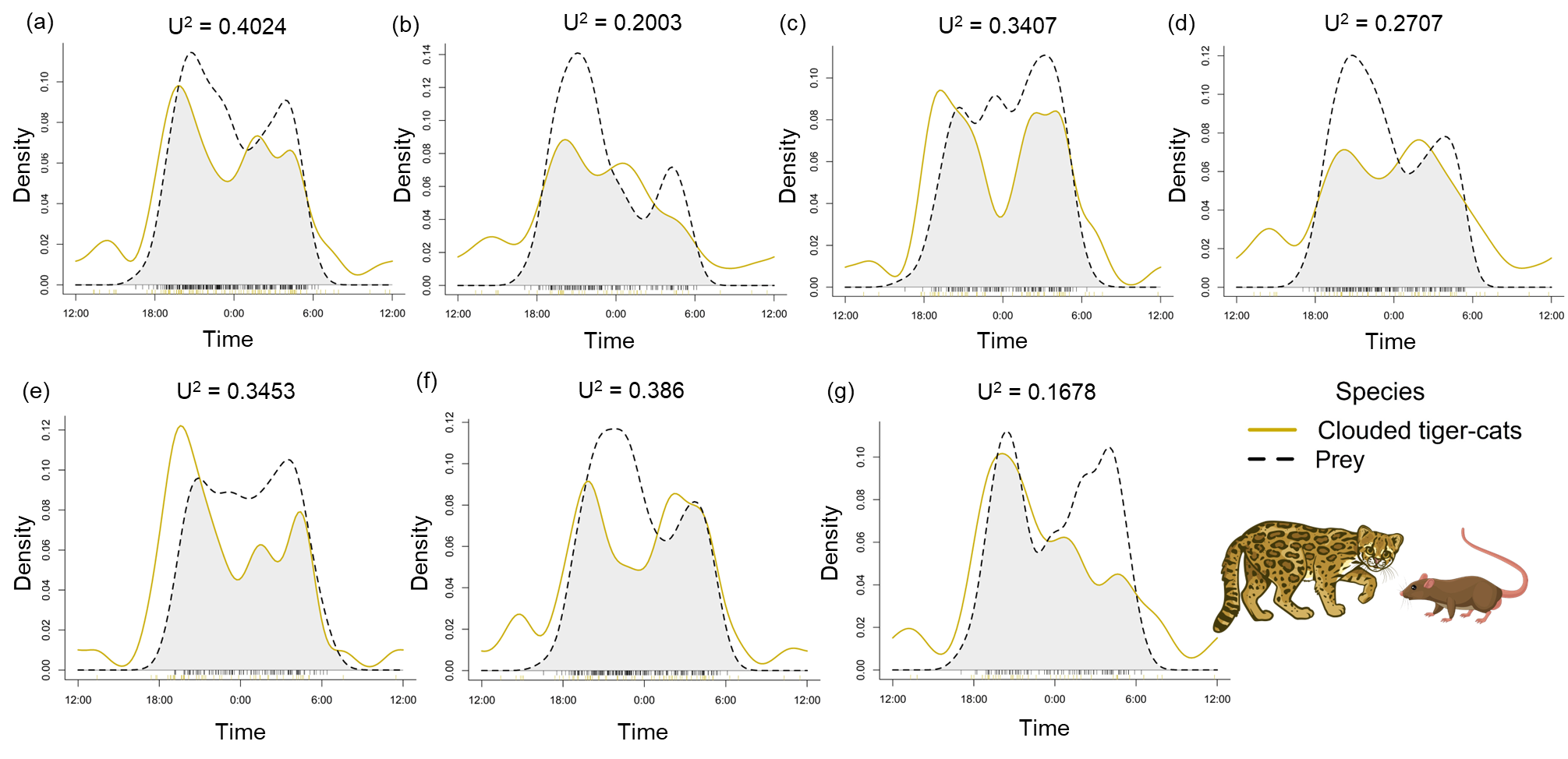


**Figure S7.** Activity curves of clouded tiger-cats and small mammal prey in the Alto del Nudo Soil Conservation District based between September 2022 and September 2023 for the full duration of the study (a), the rainy (b) and dry season (c), sites with high (d) and low (e) dog encounter rates, and human activity in the form of days of working (f) and resting (g). The shaded area is the overlap between the activity curves of both species. The Watson Two (U^2^) test are also provided. The vector image of small mammal prey was obtained from <https://www.vecteezy.com/> and of dogs was obtained from <https://www.canva.com/>.


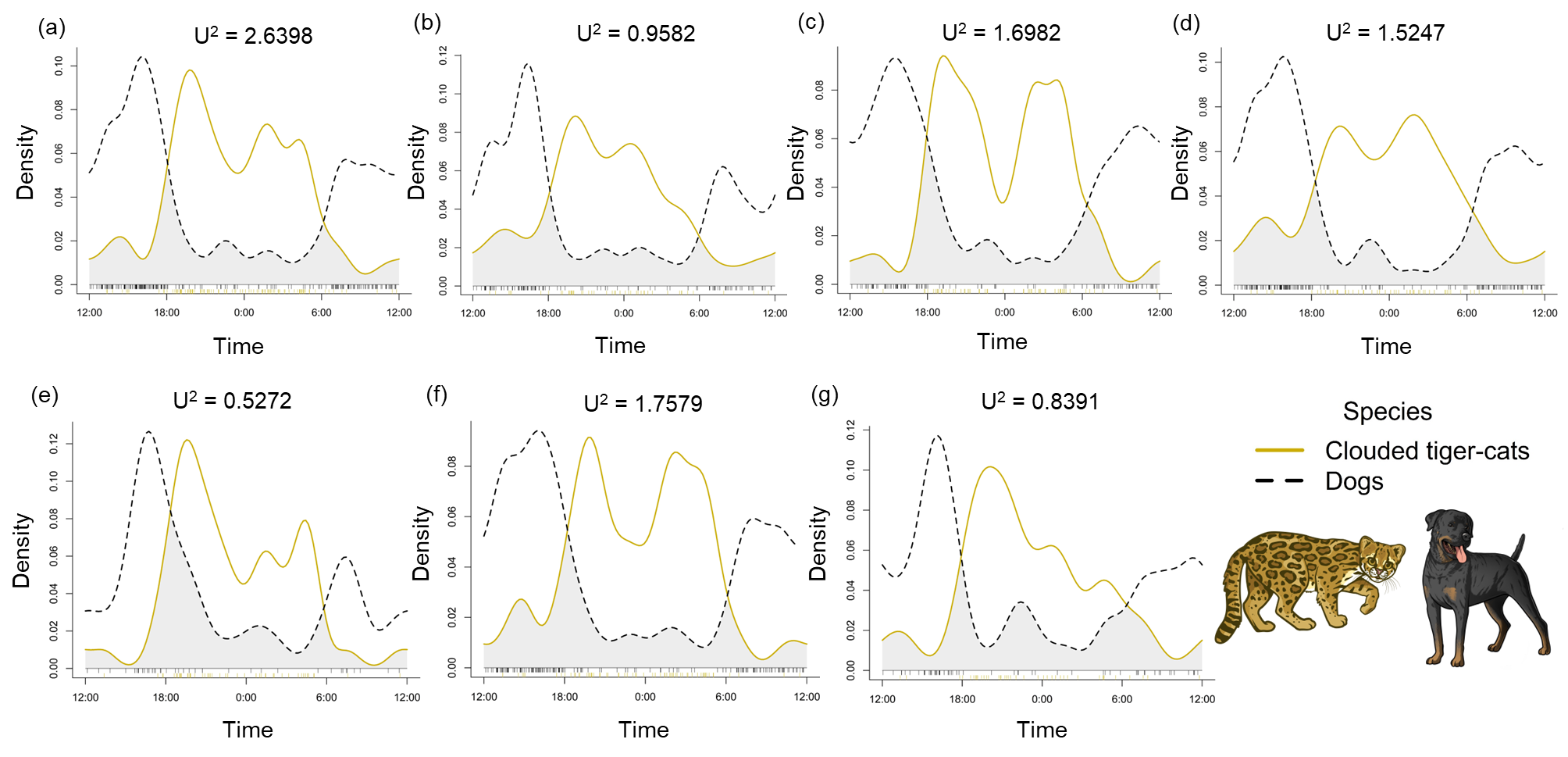


**Figure S8.** Activity curves of clouded tiger-cats and dogs in the Alto del Nudo Soil Conservation District based between September 2022 and September 2023 for the full duration of the study (a), the rainy (b) and dry season (c), sites with high (d) and low (e) dog encounter rates, and human activity in the form of days of working (f) and resting (g). The shaded area is the overlap between the activity curves of both species. The Watson Two (U^2^) test are also provided. The vector image of small mammal prey was obtained from <https://www.vecteezy.com/> and of dogs was obtained from <https://www.canva.com/>.


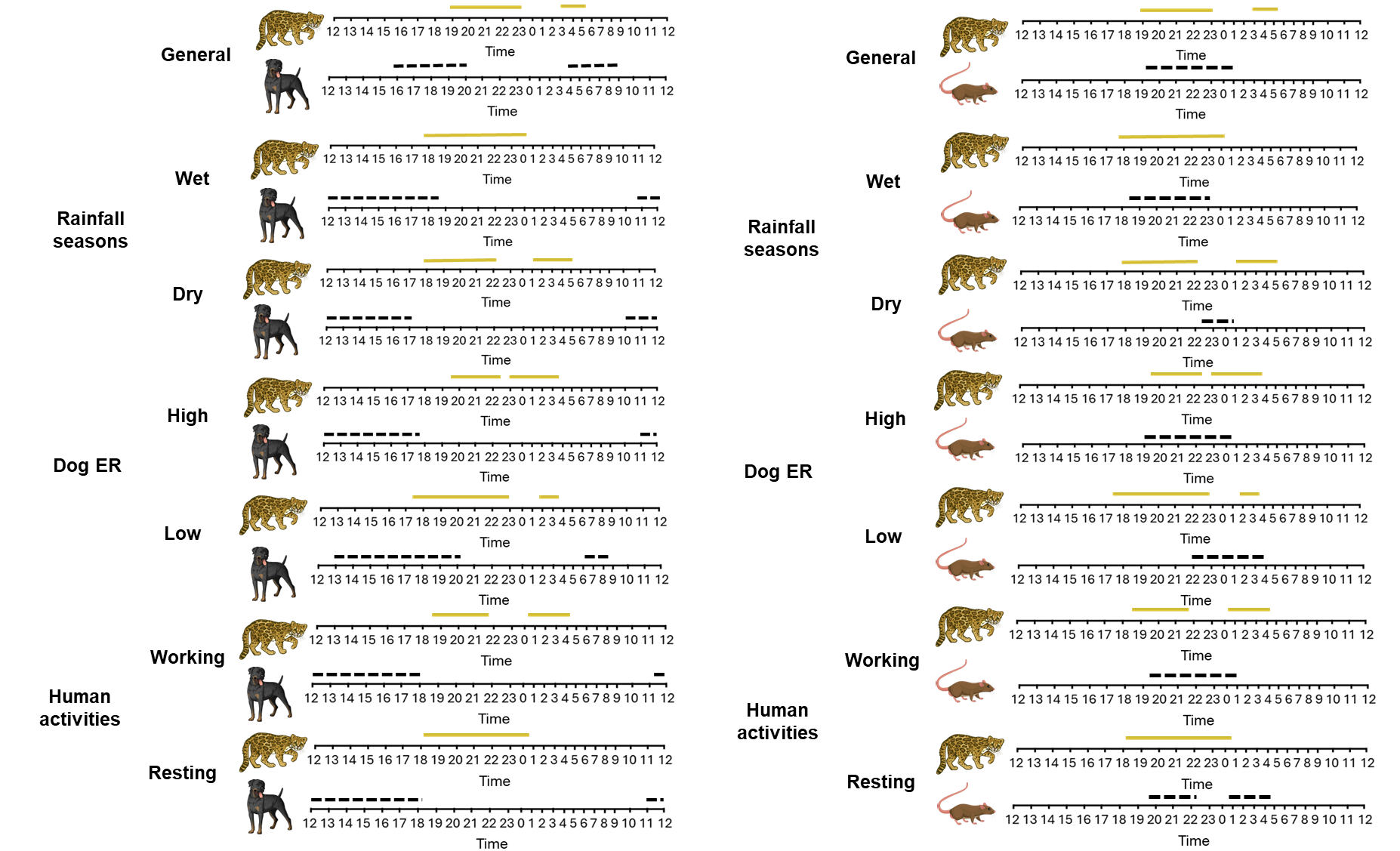


**Figure S9.** Core activity (time interval of the measured 50% isopleth or the shortest time interval containing 50% of all detections) of clouded tiger-cats, dogs and small mammal prey in general, among rainfall seasons, and. The vector image of small mammal prey was obtained from <https://www.vecteezy.com/> and of dogs was obtained from <https://www.canva.com/>.

**Table S1.** Predictors included in the negative binomial models to understand the habitat use of clouded tiger-cats and dogs for the camera trap survey conducted between September 2022 and September 2023 in the Alto del Nudo Soil Conservation District. The minimum (Min) and maximum (Max) values of each predictor, the corresponding descriptive statistics (mean, median and standard deviation - SD), and the Variance Inflation Factor (VIF) are provided. In the VIF column we indicated the resulting VIF value after dropping the colinear predictors (elevation, distance to human settlements) between parentheses.

| Covariate | Units | Min | Max | Mean (SD) | Median | VIF | Source |
| --- | --- | --- | --- | --- | --- | --- | --- |
| Sampling effort | Days | 60 | 351 | 211.5 (105.7) | 218.5 | 1.3 (1.21) | Field |
| Canopy height | Meters (m) | 5 | 27 | 15.7 (6.8) | 14 | 3.7 (1.77) | Potapov et al., 2021 |
| Canopy cover | Proportion (%) | 7 | 98 | 82.3 (26.2) | 95 | 3.3 (1.37) | Hansen et al., 2013 |
| Dog encounter rate | Number of 60-minute detections per sampling effort | 0 | 112.5 | 9.2 (8.47) | 2.6 | 3.9 (1.38) | Field |
| Small mammal encounter rate | Number of 60-minute detections per sampling effort | 0 | 34.5 | 8.7 (10.8) | 4.7 | 2.9 (1.30) | Field |
| Distance to human settlements | Meters (m) | 0 | 2.8 | 1.4 (0.7) | 1.5 | 20.8 | GIS |
| Distance to roads | Meters (m) | 0 | 2.7 | 1.6 (1.3) | 1.6 | 22.5 (3.83) | GIS |
| Elevation | Meters (m) | 1525 | 2136 | 1877.6 (161.6) | 1887 | 4.0 | Field |
| Slope | Degrees (°) | 2.1 | 26.6 | 19.8 (9.0) | 17 | 1.9 (1.66) | GIS |
| Aspect | Degrees (°) | 0 | 4.2 | 2.6 (1.7) | 2 | 3.2 (1.66) | GIS |
| Human Modification Index | Ordinal values from 0 to 1 | 0 | 97 | 0.1 (0.2) | 0.1 | 5.6 (1.76) | Theobald et al., 2025 |
| Forest Landscape Integrity Index | Ordinal values from 0 to 10 | 0 | 6.7 | 4.9 (1.7) | 5.1 | 1.9 (1.67) | Grantham et al., 2020 |

**Table S2.** Model selection to determine the best structure of the null models to understand the habitat use of clouded tiger-cats and dogs in the Alto del Nudo Soil Conservation District. We structured the null models to determine the relevance of using linear (LV) or quadratic (QV) variance functions in the negative binomial distribution, including or excluding sampling stations as random effects (RE) and a zero-inflation parameter (ZI). Df; Degrees of freedom, logLik; Log-likelihood, AICc; Akaike Information Criterion for small sample sizes, AIC_∆_; AICc difference, AICw; AIC weight. Since several null models scored an AIC_∆_ < 2, we decided to select the models with simplest structure to avoid overfitting.

| **Model** | **df** | **logLik** | **AICc** | **AIC_∆_** | **AIC_w_** |
| --- | --- | --- | --- | --- | --- |
| **Clouded tiger cat** |  |  |  |  |  |
| LV, no RE, no ZI | 2 | -60.996 | 126.6 | 0 | 0.243 |
| LV, no RE, ZI | 2 | -60.996 | 126.6 | 0 | 0.243 |
| QV, no RE, ZI | 3 | -59.786 | 126.9 | 0.28 | 0.211 |
| QV, no RE, no ZI | 2 | -62.065 | 128.8 | 2.14 | 0.083 |
| LV, RE, no ZI | 3 | -60.8 | 128.9 | 2.31 | 0.076 |
| LV, RE, ZI | 3 | -60.8 | 128.9 | 2.31 | 0.076 |
| QV, RE, ZI | 4 | -59.786 | 129.9 | 3.3 | 0.047 |
| QV, RE, no ZI | 3 | -62.065 | 131.5 | 4.84 | 0.022 |
| **Dog** |  |  |  |  |  |
| QV, no RE, no ZI | 2 | -71.434 | 147.5 | 0 | 0.401 |
| LV, RE, no ZI | 3 | -70.722 | 148.8 | 1.28 | 0.212 |
| QV,RE, no ZI | 3 | -70.862 | 149.1 | 1.56 | 0.184 |
| QV, RE, ZI | 3 | -71.434 | 150.2 | 2.7 | 0.104 |
| LV, no RE, no ZI | 2 | -73.595 | 151.8 | 4.32 | 0.046 |
| QV, RE, ZI | 4 | -70.862 | 152.1 | 4.58 | 0.041 |
| LV, no RE, ZI | 3 | -73.595 | 154.5 | 7.02 | 0.012 |
